# High-Purity Ethylene Production from Ethane/Ethylene Mixtures at Ambient Conditions by Ethane-Selective Fluorine-Doped Activated Carbon Adsorbents

**DOI:** 10.1021/acsami.4c20772

**Published:** 2025-01-25

**Authors:** Fahmi Anwar, Anish Mathai Varghese, Suresh Kuppireddy, Anastasios Gotzias, Maryam Khaleel, Kean Wang, Georgios N. Karanikolos

**Affiliations:** † Department of Chemical & Petroleum Engineering, Khalifa University, P.O. Box 127788, Abu Dhabi 127788, UAE; ‡ Center for Catalysis and Separation (CeCaS), Khalifa University, P.O Box 127788, Abu Dhabi 127788, UAE; § Renewable and Sustainable Energy Research Center, Technology Innovation Institute (TII), P.O. Box 9639, Masdar City, Abu Dhabi 9639, UAE; ∥ Institute of Nanoscience and Nanotechnology, National Center for Scientific Research Demokritos, Athens 15310, Greece; ⊥ Research and Innovation Center for CO_2_ and H_2_ (RICH), Khalifa University, P.O. Box 127788, Abu Dhabi 127788, UAE; # Food, Chemical and BioTechnology Cluster, Singapore Institute of Technology, 10 Dover Drive, Singapore 138683; ∇ Department of Chemical Engineering, University of Patras, Patras 26504, Greece

**Keywords:** paraffin-selective adsorbents, fluorine-doped activated carbon, ethane/ethylene mixtures, separation, breakthrough, Monte Carlo simulations

## Abstract

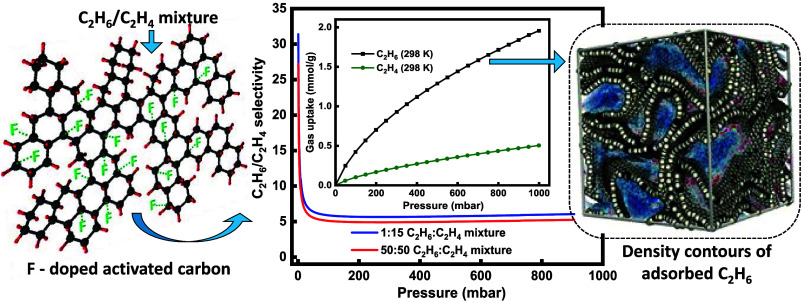

Energy-efficient separation of light alkanes from alkenes is considered as one of the most important separations of the chemical industry today due to the high energy penalty associated with the applied conventional cryogenic technologies. This study introduces fluorine-doped activated carbon adsorbents, where elemental fluorine incorporation into the carbon matrix plays a unique role in achieving high ethane selectivity. This enhanced selectivity arises from specific interactions between surface-doped fluorine atoms and ethane molecules, coupled with porosity modulation. Consequently, an equilibrium ethane/ethylene selectivity of as high as 3.9 at 298 K and 1 bar was achieved. Furthermore, polymer-grade ethylene (purity >99.99%) with a productivity of 1.6 mmol/g was obtained in a breakthrough run at ambient conditions from a binary ethane/ethylene (1/9 *v/v*) mixture. The ethane selectivity of the fluorine-doped carbons was further elucidated through Monte Carlo simulations and density contours of the adsorbed components. In addition to the high ethane selectivity, the adsorbents exhibited a hydrophobic surface, high stability under moisture, and excellent regenerability over multiple adsorption–desorption cycles under both equilibrium and dynamic conditions, demonstrating a sustainable performance.

## 1 Introduction

The separation of light olefins from undesirable paraffin molecules is considered as one of the seven most important industrial separation processes of the future in terms of market value.^1^ Considering the high energy requirement of the conventional cryogenic process for removing ethane (C_2_H_6_) from the ethylene (C_2_H_4_)-rich stream, alternative separation technologies, such as adsorption and membrane separation, are being explored.^2−4^ Carbon molecular sieve (CMS) membranes which are typically developed by pyrolysis have been reported to be promising candidates for this separation as a result of the molecular sieving action arising from their slit-like pore structures.^5^ Xu et al.^6^ further reported that incorporating ZIF-8 nanoparticles into CMS membranes during synthesis could tailor their pore structure leading to enhanced C_2_H_4_/C_2_H_6_ selectivity and C_2_H_4_ permeability. Adsorptive separation of C_2_H_6_ from C_2_H_4_-rich streams is also being investigated by a range of adsorbents, such as metal–organic frameworks (MOFs), carbon, and silica adsorbents.^7^

The majority of the developed adsorbents exhibit an ethylene selectivity. For example, metal ion-doped MOFs have been reported to exhibit high C_2_H_4_/C_2_H_6_ selectivity, such as in the case of Cu-chelated UiO-66-(COOH)_2_ with a benchmark selectivity of 80 at 298 K and 1 bar.^8^ Silver-ion-impregnated MOF Ag/CPL-2 exhibited a C_2_H_4_/C_2_H_6_ selectivity of 63 at 298 K and 1 bar, owing to the enhanced interactions between the Ag^+^ ions and C_2_H_4_ molecules.^9^ Equilibrium interactions with C_2_H_4_ because of the π-complexation phenomenon combined with kinetic separation capability enabled phosphorus–zinc-based MOF ZnAtzPO_4_ to yield an equilibrium-kinetic combined C_2_H_4_/C_2_H_6_ selectivity of 32.4, estimated based on the Henry constant and kinetic diffusivity for both gases at 273 K and 1 bar,^10^ while transient breakthrough experiments with a mixture of C_2_H_4_/C_2_H_6_ (50/50, *v/v*) demonstrated a difference of 44 min in between the elution of the two gases. Yet, the outstanding selectivity performance of the metal-doped MOFs is often surpassed by the framework instability and charge imbalance.^3^ Unlike MOFs, metal-doped carbonaceous adsorbents exhibit higher stability along with adequate olefin/paraffin separation capability. Ag^+^-doped furfuryl alcohol-based activated carbon (AC), for instance, showed a C_2_H_4_/C_2_H_6_ selectivity of 4.5 owing to the *π–π* complexation.^11^ CMK-3-ordered mesoporous carbon, when modified with CuCl by thermal dispersion, exhibited a notable increase in C_2_H_4_/C_2_H_6_ selectivity from 0.93 to 2.67 ascribed to the presence of Cu^+^ ions inside the pores.^12^

Despite the performance achieved by C_2_H_4_-selective adsorbents, the fact that the industrial feed is rich in C_2_H_4_ with traces of C_2_H_6_ demands for multiple adsorption–desorption cycles, large equipment and bed volume, and higher energy consumption in order to obtain polymer-grade C_2_H_4_ purity when C_2_H_4_-selective adsorbents are involved.^13^ To this extent, ethane-selective adsorbents are being explored, such as MOFs with aromatic ligands,^14^ and biomolecule-derived carbon adsorbents.^15^ In the case of MOFs ssuch as JXNU-9,^16^ Cu(Qc)_2_,^17^ and PCN-245,^18^ the presence of aromatic rings inside the pores facilitates C–H···π interactions with the C_2_H_6_ molecules. Structurally and chemically stable AC adsorbents have also been reported to show high C_2_H_6_ capacity though the C_2_H_6_/C_2_H_4_ selectivity of such absorbents, unlike MOFs, is relatively low. C_2_H_6_ molecules exhibit higher polarizability than C_2_H_4_ molecules (44.7 × 10^–25^ versus 42.52 × 10^–25^ cm^3^, respectively);^19,20^ consequently, carbonaceous adsorbents were reported to be ethane-selective arising from the surface–adsorbate interactions with the heavier C_2_H_6_ molecules. Furthermore, the spatial limitations in enabling interactions with the carbon-rich surface for the sp^2^ hybridized C_2_H_4_ molecules compared to the sp^3^ hybridized C_2_H_6_ ones result in selective uptake of the latter.^21^ The enhanced van der Waal’s interactions between the C_2_H_6_ molecules and the carbon surface is the main fundamental mechanism behind the ethane selectivity in carbonaceous adsorbents such as Glc-As-4,^15^ MGA-700-3,^22^ and C-Fru-4-700.^23^

Although several carbonaceous adsorbents have demonstrated notable C_2_H_6_ capacity and selectivity, their selectivity still remains too low to be applied at an industrial scale and meet the produced olefin purity and productivity targets. The interaction between C_2_H_6_ molecules and a carbon surface can be improved through surface modification by using various functional agents. Studies have demonstrated that physical impregnation or covalent grafting of molecules with an affinity for C_2_H_6_ can enhance the interaction between C_2_H_6_ molecules and the adsorbent surface. Indeed, modification of the UiO-67 MOF with –NH_2_ groups resulted in an increment in C_2_H_6_/C_2_H_4_ selectivity from 1.5 to 1.7 and an increase in C_2_H_6_ capacity from 3 to 5.3 mmol/g at 298 K and 1 bar.^24^ The presence of electronegative fluorine atoms in the pore walls enhanced C_2_H_6_···adsorbent affinity for ZU-36-Ni,^25^ UPC-104,^26^ and FMOF-2^27^ arising from C–H···F interactions. On the basis of the above advancements, the effect of fluorine atoms as ethane selectivity enhancers, and the stability and robustness of carbon adsorbents, we synthesized here a series of fluorine-doped AC adsorbents exhibiting ethane selectivity for the separation of C_2_H_6_/C_2_H_4_ gas mixtures. Fluorine-doped-activated carbon offers a unique combination of enhanced ethane selectivity, cost-effectiveness, structural stability, and environmental robustness, making it highly attractive for industrial gas separations. Fluorine-functionalized ethane-selective adsorbents with excellent performance and stability were developed from cost-effective activated carbon, achieving enhanced selectivity (3.9 for AC-F-20 at 298 K and 1 bar), surpassing the performance of similar carbon-based adsorbents in C_2_H_6_/C_2_H_4_ separation under comparable conditions. Detailed parametric investigation, characterization, and performance evaluation were conducted to identify the optimal conditions to yield polymer-grade ethylene under ambient conditions and the underlining adsorption mechanisms, while Monte Carlo simulations corroborated the experimental findings, further validating the consistency of the results and aiding in optimizing the adsorbent configurations.

## 2 Experimental Section

### 2.1 Materials

Nuchar activated carbon (AC) was obtained from Ingevity. Sodium fluoride (NaF, 99.99%) and ethanol (C_2_H_6_O, 99%) from Sigma-Aldrich, and sulfuric acid (H_2_SO_4_, 97%) from Merck were used for the synthesis of the adsorbents. N_2_ (99.99%) and He (99.99%) gases from Air Products, and C_2_H_6_ (99.95%), C_2_H_4_ (99.99%), and a C_2_H_6_/C_2_H_4_ (1/9, *v/v*) mixture from Gulf Cryo were used for the adsorption experiments.

### 2.2 Synthesis of Fluorine-Functionalized AC

Modification of the AC was carried out as reported by Kakaei et al.^28^ In a particular example, the fluorine solution was first prepared by dissolving 5 mg of NaF in 15 mL of 10 M H_2_SO_4_. Subsequently, 250 mg of AC was added to the acid solution and sonicated for 30 min. The reaction mixture was stirred overnight at 25 °C. The final product was then filtered and washed multiple times with DI water and ethanol and dried afterward at 80 °C. The adsorbent was activated by heating in a tube furnace under a N_2_ atmosphere at 300 °C for 2 h. By variation of the amount of NaF in the reaction mixture, a range of AC samples with different fluorine loadings were synthesized as listed in Table S1.

### 2.3 Characterization

X-ray diffraction (XRD) patterns were obtained using a Bruker D2 Phaser diffractometer in the 2θ range of 10–50° with a scan rate of 0.05 °s^–1^ (45 kV, 40 mA). Fourier transform infrared (FTIR) spectra were acquired using a Bruker Tensor II in Attenuated total reflectance (ATR) mode in the wavenumber range of 500–4000 cm^–1^. A PerkinElmer 4000 Thermogravimetric Analyzer (TGA) was used to study the thermal stability of the synthesized samples by heating approximately 5 mg of sample at a rate of 5 °C/min under N_2_ from 50 to 600 °C. Scanning electron microscopy (SEM) images were obtained at 15 kV on an FEI Nova Nano SEM 650. Elemental composition was determined by energy dispersive X-ray spectroscopy (EDS) at five different sample locations and then taking the numerical average. A Witec α 300 RAS was used to acquire Raman spectra in the range 500–3000 cm^–1^ with a laser wavelength of 532 nm. A Micromeritics 3Flex Analyzer was used to perform N_2_ adsorption–desorption experiments at 77 K, moisture uptake analysis at 298 K, and equilibrium adsorption experiments with C_2_H_6_ and C_2_H_4_ at 298, 273, and 263 K, and up to 1 bar. The surface area was determined using the Brunauer–Emmett–Teller (BET) model, while the mesopore size distribution was calculated using the Barrett–Joyner–Halenda (BJH) model. The BET analysis utilized isotherm data within the pressure range of 0.05–0.35 (*p*/*p*_o_). The adsorbents were pretreated at 100 °C for 12 h prior to the adsorption experiments. An automated breakthrough analyzer (ABR) by Hiden Isochema was utilized to carry out dynamic breakthrough experiments with C_2_H_6_/C_2_H_4_ mixtures. The reactor column (15 cm × 1 cm) was filled with the preweighed sample and, before each breakthrough run, helium was purged at a rate of 250 mL/min. Then a mixture of C_2_H_6_/C_2_H_4_ (1/9, *v/v*) was passed at a rate of 5 mL/min, while the reactor conditions were kept at 298 K and 1 bar. The composition of the mixture leaving the column was analyzed using a single-filter, electron multiplier dynamic sampling mass spectrometer (DSMS) by Hiden, with a detection range of 1–200 AMU and a sensitivity of 100 ppb.

### 2.4 Molecular Modeling

In the Monte Carlo simulations, we used an atomically detailed model of activated carbon (aCF) consisting of 32,000 carbon atoms. This model, developed by Marks and his collaborators,^29^ was sourced from a publicly accessible database of amorphous carbon models provided by Thyagarajan and Sholl,^30^ where it is listed as aCarbon-Marks-id014. To decorate the carbon sample with fluorine atoms, a specific number of randomly selected carbon atoms in the framework were replaced with fluorine. This process resulted in the creation of three fluorine-doped activated carbons, designated as aCF2, aCF4, and aCF6, corresponding to the substitution of 640 (2%), 1280 (4%), and 1920 (6%) carbon atoms with fluorine, respectively.

The frameworks were modeled as rigid structures within a cubic unit cell with a cell vector of 89.16 Å, and periodic boundary conditions were applied in all directions. The simulated samples display high porosity with significant volume fractions, and their structural parameters are detailed in Table 1. The framework density is determined by dividing the molar mass of the framework atoms by the unit cell volume. The helium void fraction is calculated by inserting a probe atom into the cell and counting the instances where it experiences a negative interaction potential (i.e., no overlap with framework atoms). The probe atom is modeled as helium, with ε = 10.9 K and σ = 2.64 Å. The available pore volume is then obtained by multiplying the unit cell volume by the helium void fraction.

**Table 1:** Structural Parameters of the Carbon Models Used (Where aC Represents the All-Carbon Structure, While aCF2, aCF4, and aCF6 Contain 2, 4, and 6% Fluorine Atoms, Respectively); Each Structure Consists of a Total of 31,896 Atoms, and the Cubic Unit Cell Has a Vector Length of 89.16 Å

structural parameter	aC	aCF2	aCF4	aCF6
framework density (g/cm^3^)	0.896	0.907	0.917	0.927
helium void fraction	0.311	0.307	0.311	0.312
available pore volume (cm^3^/g)	0.345	0.342	0.339	0.337
fluorine atoms (%)	0 (0%)	640 (2%)	1280 (4%)	1920 (6%)

Ethane was modeled as two CH_3_ monomers connected by a harmonic bond, while ethylene was represented as a rigid, linear molecule composed of two bonded CH_2_ monomers. The CH_3_ and CH_2_ monomers were treated as united atoms with Lennard–Jones parameters of ε = 108.0 K and σ = 3.76 Å for CH_3_, and ε = 56.0 K and σ = 3.96 Å, for CH_2_.^31^ The DREIDING force field was applied for the interactions of the framework, with Lennard–Jones parameters of ε = 47.85 K and σ = 3.47 Å for carbon atoms, and ε = 36.48 K and σ = 3.09 Å for fluorine atoms.^32^ The general Lorentz–Berthelot mixing rule was applied to all intermolecular interactions with a cutoff radius of 12 Å.

To visualize the density contours of C_2_H_6_ and C_2_H_4_ adsorption, we constructed a 3D histogram of the adsorbate atomic positions within the unit cell was constructed. The unit cell was divided into 150 × 150 × 150 finite volumes. During the grand-canonical Monte Carlo simulations, the pressure was set to 1 bar and the temperature to 298 K, with the adsorbate molecules allowed to translate, rotate, and be deleted with equal probability. A total of 10^6^ cycles were run, with the first 20,000 cycles disregarded to account for equilibration. As the molecules moved through the cell, data for the histogram was collected at each simulation cycle. The position of each molecule was mapped to its corresponding finite volume in the grid, increasing the index for that element. The histogram was periodically saved in a file for subsequent visualization. The simulations were conducted using RASPA,^33^ and the visualizations were produced using ParaView.^34^

The Henry coefficient represents the slope of the adsorption isotherm at low adsorbate loadings. To determine the Henry coefficients for C_2_H_6_ and C_2_H_4_ adsorption in both unmodified and fluorinated AC samples, the Widom test particle insertion method^35^ was used at the desired temperature. In this approach, a single adsorbate molecule was randomly inserted into the unit cell and the energy of its interaction with the solid framework was computed. This energy is linked to the chemical potential of (not too dense) adsorbate systems and the enthalpy of adsorption at the limit of zero coverage, which, in turn, correlates to the Henry coefficient. A total of 10^6^ Widom insertion cycles were performed, using either an ethane or ethylene molecule as test particles.

## 3 Results and Discussion

### 3.1 Adsorbent Characteristics

**Figure 1:**
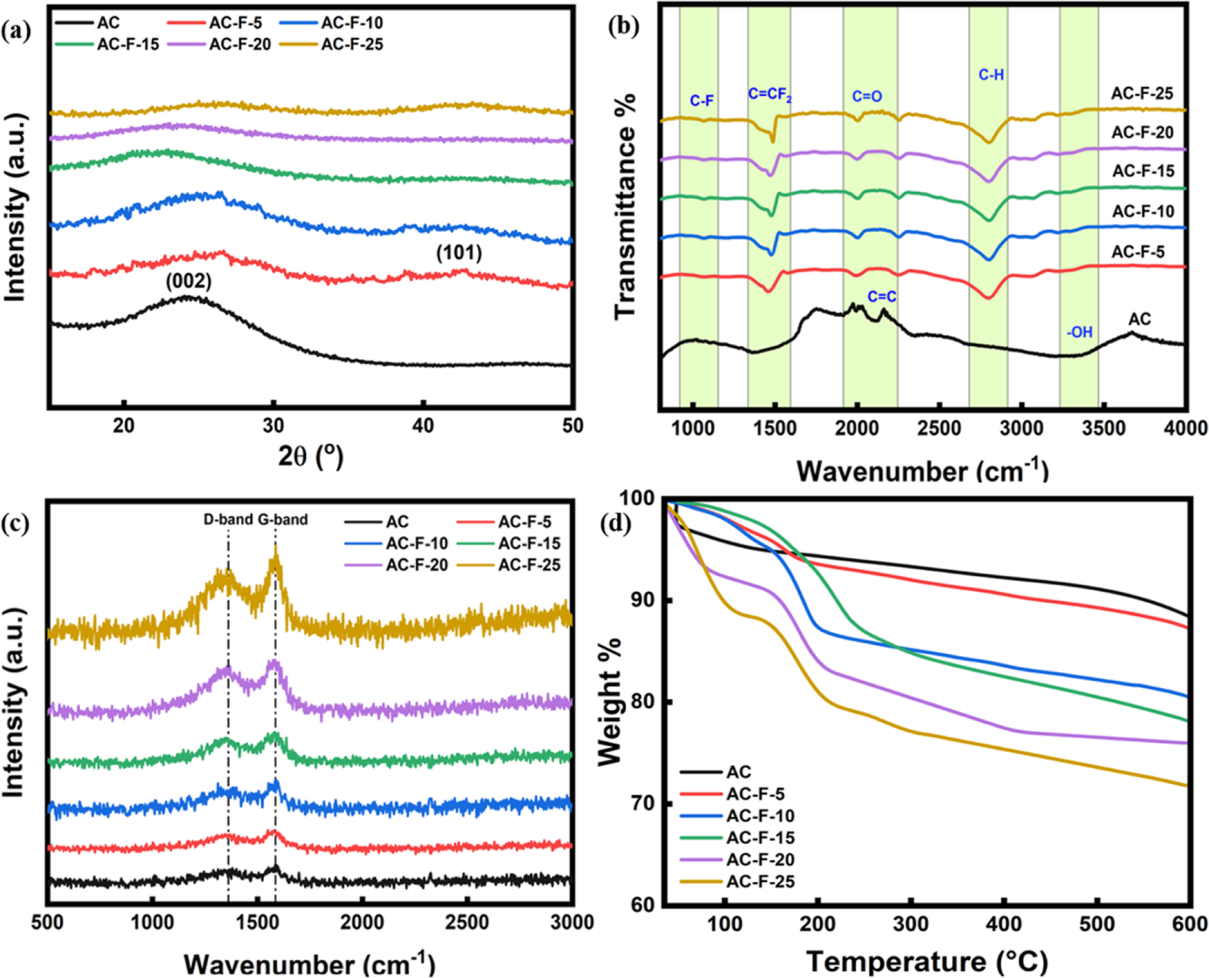
(a) XRD patterns, (b) FTIR spectra, (c) Raman spectra, and (d) TGA profiles of the pristine and fluorine-modified AC adsorbents.

The XRD patterns of the prepared adsorbents are shown in Figure 1a. The pristine AC exhibited a broad diffraction peak at 26°^36^ assigned to the (002) plane of the carbon matrix indicative of short-range ordering of graphitic layers within the amorphous structure. Upon fluorine doping, the position of this peak was shifted slightly as a result of a change in the lattice structure. The fluorine atoms, which are smaller than the carbon ones, when incorporated into the carbon matrix, distorted the lattice planes, resulting in the peak shifting. For the samples AC-F-5, AC-F-10, and AC-F-25, a wide diffraction peak was also observed at 44° corresponding to the (101) plane, formed as a result of the occurrence of new crystalline-like phases. It is therefore evident that such doping causes alteration of the carbon matrix structure, leading to the formation of new planes and change in the lattice parameters of existing planes.^37,38^ FTIR spectroscopy was employed to comprehend the change in the functional groups present on the carbon surface upon fluorine doping. As illustrated in Figure 1b, hydroxyl O–H stretching (3344 cm^–1^), C–O stretching (1408 cm^–1^), carbonyl and carboxyl C=O stretching (1904 cm^–1^), alkenyl C=C stretching (2114 cm^–1^), and skeletal C–C stretching (1190 cm^–1^) were detected in the pure AC.^39,40^ For the functionalized samples, polarized C–F bond stretching (1067 cm^–1^) of the tertiary carbon atom and asymmetric stretching of C=CF_2_ (1485 cm^–1^) groups located at the edges were identified, confirming the fluorination.^41,42^ Furthermore, the peak for aliphatic C–H (2800 cm^–1^)^43^ stretching was also identified for the fluorinated samples.

Figure 1c shows the Raman spectra of the synthesized adsorbents. All samples display two prominent peaks of the D and G bands at 1362 and 1585 cm^–1^, respectively. The D band represents the disorder and lattice defects in the structure, whereas the G band corresponds to the ordered sp^2^ hybridized carbon atoms.^44^ The disorder in the adsorbent structure after fluorination can be assessed by the intensity ratio of the D band to the G band (*I*_D_/*I*_G_ ratio), where a smaller *I*_D_/*I*_G_ ratio indicates a more ordered and symmetric carbon matrix.^45^ With an increase in the degree of fluorination, the D band intensity increased with a corresponding increment in the *I*_D_/*I*_G_ ratio as well, specifically from 0.97 for AC-F-5 to 0.99 of AC-F-25.^36^ This increase in the D band intensity is attributed to the introduction of defects and disorder in the carbon lattice due to the incorporation of the fluorine groups. The fluorine groups can break the symmetry of the carbon lattice, leading to the formation of new sp^3^ hybridized carbon atoms and the creation of defect sites. These newly formed lattice defects can function as selective adsorption sites.

Figure 1d displays the TGA profiles of the adsorbents. The pristine AC exhibited a weight loss step corresponding to moisture removal up to 120 °C, followed by decomposition of the carbon skeleton starting at 500 °C. On the other hand, the weight loss for the fluorine-modified samples occurred in three stages. The initial stage was due to the loss of solvent and moisture, followed by the decomposition of organic impurities, breaking of C–F bonds, and the release of C_2_F_6_ at 150–200 °C.^46^ Finally, the weight loss above 500 °C was attributed to the decomposition of the carbonaceous matrix.^39^ For the fluorinated samples, the weight loss increased with the fluorination degree.

**Figure 2:**
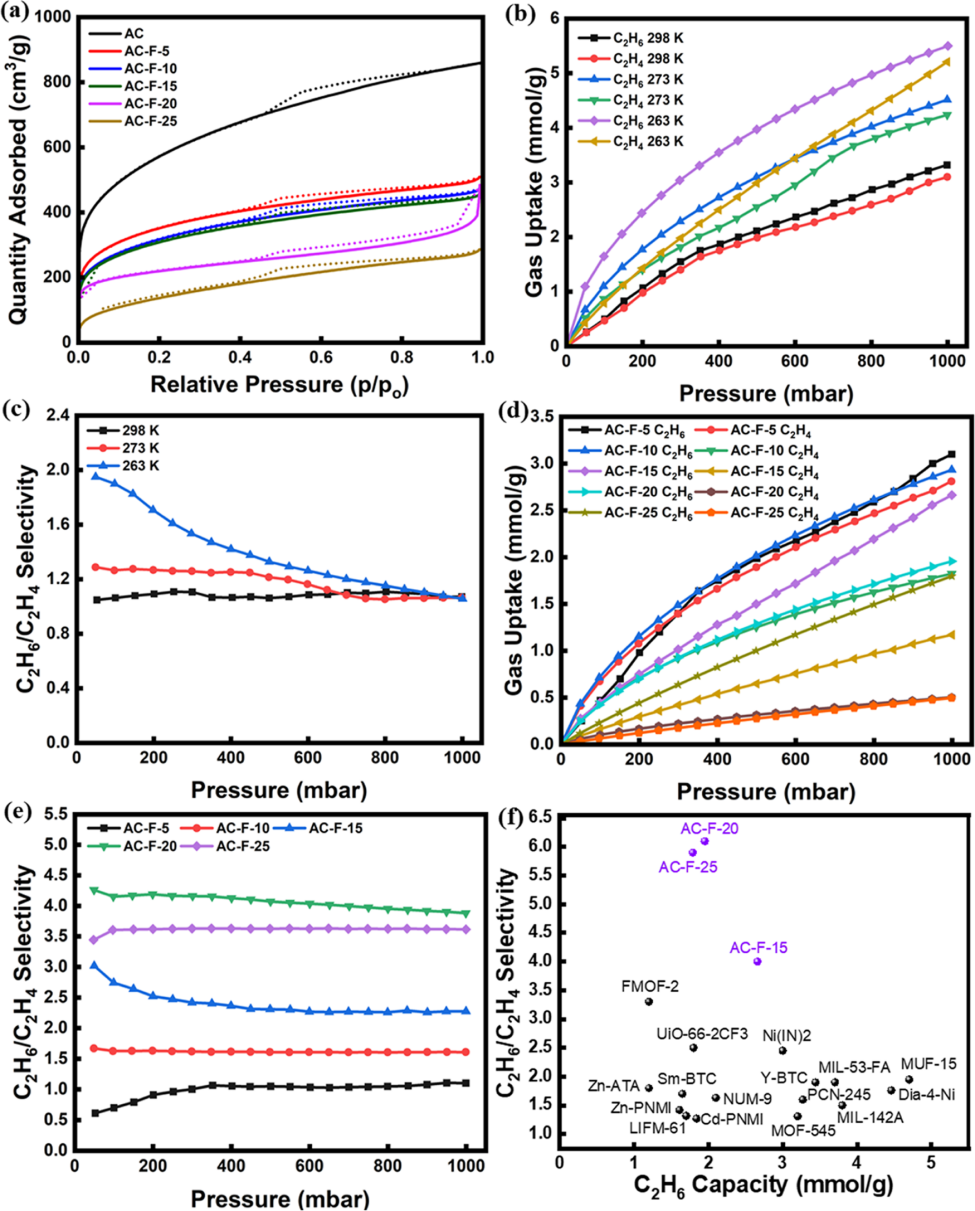
(a) N_2_ adsorption/desorption isotherms at 77 K for the pure and fluorine-modified AC samples. (b) C_2_H_6_ and C_2_H_4_ adsorption isotherms and (c) experimental C_2_H_6_/C_2_H_4_ selectivity for pristine AC at three temperatures (298, 273, and 263 K). (d) C_2_H_6_ and C_2_H_4_ adsorption isotherms and (e) experimental C_2_H_6_/C_2_H_4_ selectivity for the fluorine-modified adsorbents at 298 K. (f) Comparison plot of C_2_H_6_/C_2_H_4_ selectivity vs C_2_H_6_ capacity for C_2_H_6_-selective adsorbents (the references for the corresponding adsorbents are given in Table S3).

To evaluate the textural characteristics of the adsorbents, N_2_ adsorption–desorption isotherms were collected at 77 K (Figure 2a). The acquired isotherms of the samples were observed to be closer to type I with a small hysteresis in the higher *p*/*p*_o_ range, indicating a microporous nature with the presence of some mesopores. The micropore size distribution, estimated by the Horvath–Kawazoe (HK) model^47^ and shown in Figure S1, further supports the predominance of microporosity in the samples. In the fluorine-doped materials, the surface area as well as pore volume decreased, as shown in Table 2. Indeed, the BET surface area of the pristine AC was 1983 m^2^/g, which decreased with increasing fluorine loading to 1220, 1102, 1071, 775, and 448 m^2^/g for AC-F-5, AC-F-10, AC-F-15, AC-F-20, and AC-F-25 respectively. Likewise, pristine AC had a total pore volume of 1.33 cm^3^/g which decreased to 0.44 cm^3^/g for AC-F-25. This is attributed to partial blockage of the pores by the fluorine atom incorporation, while the increase in average pore size with the degree of functionalization indicates that the smaller micropores undergo complete blocking, in agreement with the reduction in N_2_ uptake at the very low *p*/*p*_o_ range.

**Table 2:** Textural and Elemental Data of AC and the Modified AC Samples

sample	*S*_BET_ (m^2^/g)	total pore volume (cm^3^/g)	average pore size (nm)	C atomic %	O atomic %	F atomic %
AC	1983	1.33	2.68	92.94	7.06	
AC-F-5	1220	0.79	2.58	90.36	9.30	0.34
AC-F-10	1102	0.73	2.65	88.34	10.04	1.62
AC-F-15	1071	0.71	2.63	87.30	10.36	2.34
AC-F-20	775	0.65	3.87	82.08	11.73	6.19
AC-F-25	448	0.44	3.95	79.86	10.37	9.77

The morphological features of the adsorbents were investigated by SEM. Based on Figure S2, it is observed that the AC samples consist of particles/crystals with heterogeneous sizes and shapes^48^ The elemental composition of the samples were assessed by EDS (Table 2). With increasing fluorine doping, the atomic % of fluorine present in the samples increased, confirming the presence of fluorine in the adsorbents and verifying the increasing degree of functionalization. Indeed, the F/C ratio increased from 0.004 for AC-F-5 to 0.122 for AC-F-25. Interestingly, the oxygen content also increased on the modified samples, indicating that F-doping promoted activation of the carbon that increased the reactivity of the latter with residual oxygen so that more heterogeneous structures were produced that host both F- and O- functional groups.^49^ Since the O- also presents a strong electronegativity, it is anticipated to also play a role in enhancing the ethane selectivity.

### 3.2 Equilibrium Adsorption Evaluation

We first evaluated the single-component equilibrium adsorption of C_2_H_6_ and C_2_H_4_ on pristine AC at various temperatures, i.e., 298, 273, and 263 K, and up to 1 bar (Figure 2b). AC exhibited high gas adsorption capacity across all three temperatures, which can be attributed to its high surface area and microporosity, as verified by N_2_ isotherm analysis. Yet, at 298 and 273 K, despite the high gas uptakes, the C_2_H_6_ uptake was only slightly higher than that of C_2_H_4_. As a result, the experimental C_2_H_6_/C_2_H_4_ selectivity at 1 bar was 1.1 and 1.2, respectively. At 263 K, the C_2_H_6_ uptake was substantially higher than that of C_2_H_4_, particularly in the lower pressure range. Yet, as the pressure increased to 1 bar, the adsorption capacities of both gases became similar. It should also be noted that, despite the fact that the selectivity was higher at 263 K, operation at such low temperature is not a viable option as substantial energy needs to be supplied for cooling.

Figure 2d shows the adsorption isotherms of the modified AC samples at 298 K. AC-F-5, which was the adsorbent with the lowest fluorine loading, exhibits a C_2_H_6_ capacity of 3.1 mmol/g at 1 bar which was reduced to 2.9, 2.66, 1.95, and 1.79 for AC-F-10, AC-F-15, AC-F-20, and AC-F-25 respectively, demonstrating a gradual decline of C_2_H_6_ uptake with increasing fluorine content. This trend is attributed to the reduction in the adsorbent surface area and pore volume, as observed from the N_2_ isotherms discussed above. In contrast to the capacity values, the experimental selectivity followed the opposite trend, i.e., it increased from 1.1 for AC-F-5 to 3.9 for AC-F-20 at 1 bar, owing to the increasing density of the functional agents and the associated effect on surface and porosity merits. Afterward, it stabilized, and even slightly decreased to 3.6 for AC-F-25, which is attributed to the fact that overloading with functional agents on the surface does not further increase the specific sites, while it decreased the effective surface area for adsorption leading to a decrease in selectivity for AC-F-25.^3^ The significant increase in C_2_H_6_/C_2_H_4_ selectivity for the fluorine-doped adsorbents (from 1.1 for pure AC to 3.9 for AC-F-20 at 298 K and 1 bar) is attributed to the C–H···F interactions between the C_2_H_6_ molecules and the fluorine atoms present on the surface and the pore structure alteration due to the incorporation of the fluorine atoms that generates additional specific adsorption sites. With respect to the former in particular, C_2_H_6_ can form six such hydrogen bonds compared to four of C_2_H_4_, and these interactions were found to be crucial in enhancing the C_2_H_6_ selectivity.^50^

**Figure 3:**
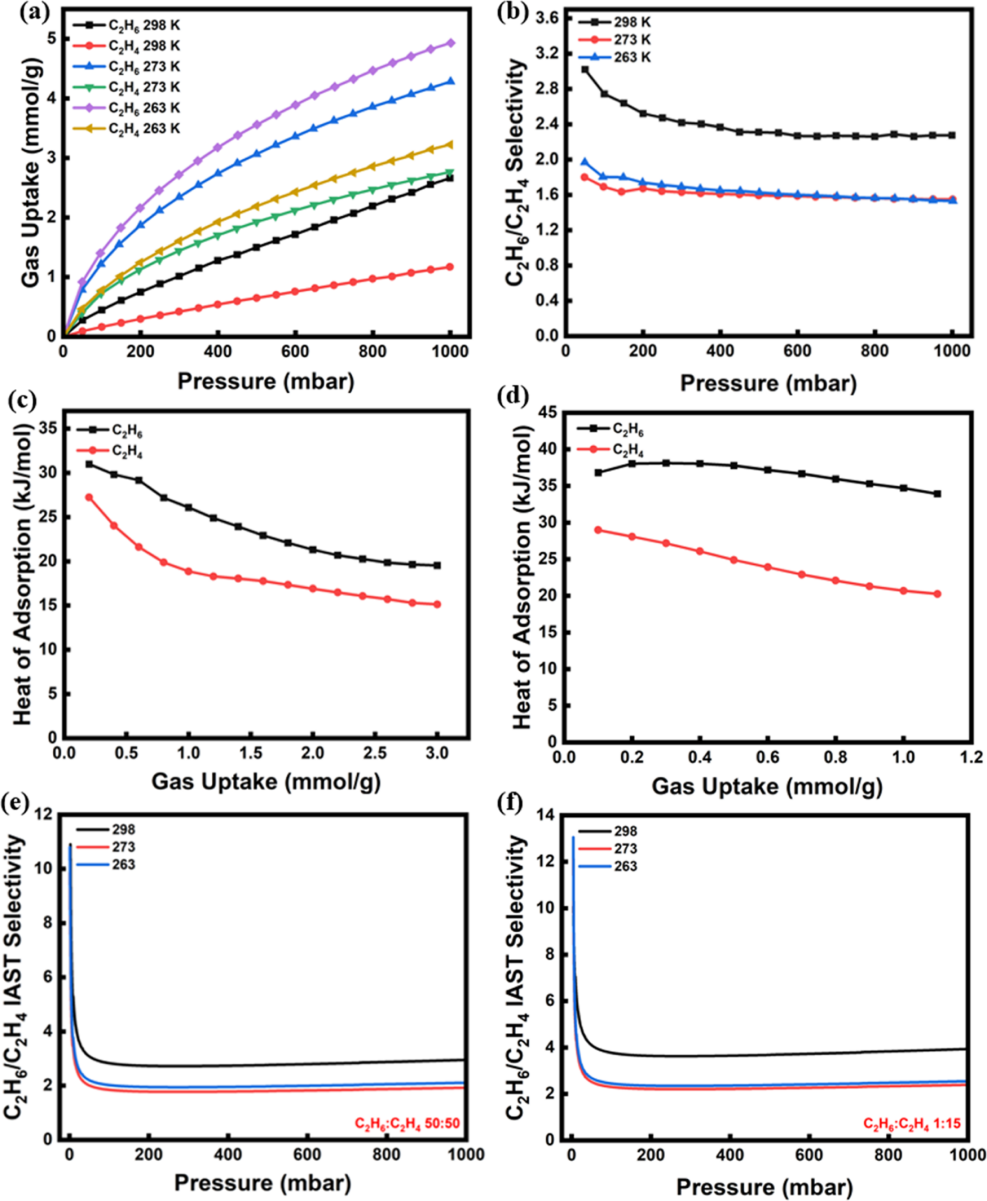
(a) C_2_H_6_ and C_2_H_4_ adsorption isotherms and (b) C_2_H_6_/C_2_H_4_ experimental selectivity of AC-F-15 at three temperatures (298, 273, and 263 K), isosteric heat of adsorption for (c) AC and (d) AC-F-15, and C_2_H_6_/C_2_H_4_ IAST selectivity for (e) 50/50 (*v/v*) and (f) 1/15 (*v/v*) mixture of C_2_H_6_:C_2_H_4_ for AC-F-15.

Based on the above evaluation, AC-F-15 and AC-F-20 were deemed to be optimal samples with respect to selectivity and capacity; thus, they were subject to further investigation. AC-F-15 demonstrated a C_2_H_6_ uptake of 2.7 mmol/g at 298 K and 1 bar, which compared favorably to the C_2_H_4_ uptake of 1.2 mmol/g at the same conditions. At lower temperatures (Figure 3a), the C_2_H_6_ uptake for the same sample increased (4.3 and 4.9 mmol/g at 273 and 263 K, respectively) indicating that physisorption is retained as the main governing mechanism of adsorption in the modified samples as well and revealing that no strong binding takes place between the C_2_H_6_ molecules and the fluorine-doped surface. Similarly, the C_2_H_4_ uptake increased to 2.7 and 3.2 mmol/g under the same conditions, respectively. However, the highest selectivity was observed at 298 K (2.8 at 50 mbar and 2.3 at 1 bar), while at 273 and 263 K, a selectivity of 1.5 at 1 bar was attained. Overall, we can anticipate two types of adsorption sites/mechanisms on the modified samples: physical adsorption in the pores, and adsorption onto doped sites largely based on van der Waal’s interactions. While the former is less-selective and temperature sensitive, the latter is more-selective and less temperature sensitive; therefore, higher selectivity is observed at higher temperatures. Furthermore, at lower temperatures, partial condensation of the hydrocarbon gases in pores smaller than 1 nm may occur, resulting in suppression of the specific interactions between the adsorbent surface and C_2_H_6_ molecules.^51^

To further evaluate the nature of interactions between the adsorbent surface and adsorbate gases, the isosteric heat of adsorption (*Q*_st_) was determined. The adsorption isotherms at three different temperatures, specifically at 298, 273, and 263 K, were fitted using the dual-site Langmuir (DSL)^52^ model (eq S1), to estimate the isosteric heat of adsorption (eq S2)^53^ for both C_2_H_6_ and C_2_H_4_. The corresponding fitting parameters are listed in Table S2. The plots of the heat of adsorption vs gas loading for AC and AC-F-15 are shown in Figure 3c,d, respectively. At a loading of 1 mmol/g, AC had *Q*_st_ values of 26 and 19 kJ/mol for C_2_H_6_ and C_2_H_4_, respectively. The heat of adsorption for both gases decreased with loading, indicating decrease in the strength of the adsorption sites and progressively diminishing interactions between the surface and gas molecules as the adsorption progresses. However, for the modified adsorbent, AC-F-15, the stronger interactions between the doped surface and the paraffin molecules resulted in a higher *Q*_st_ value for C_2_H_6_ of 35 kJ/mol at 1 mmol/g, which justifies the observed enhanced selectivity. In addition, the heat of adsorption for C_2_H_6_ exhibited a nearly constant value with loading indicating steady affinity and uniform interactions of the adsorption sites with the C_2_H_6_ molecules. For C_2_H_4_, on the other hand, *Q*_st_ did not change considerably upon fluorination, while it kept decreasing with gas loading, with a value of 20 kJ/mol at 1 mmol/g for AC-F-15, which signifies the preferential interaction of the fluorine-functionalized surface with the C_2_H_6_ molecules.

The selectivity based on the ideal adsorbed solution theory (IAST) was also estimated for the AC-F-15 adsorbent at different temperatures (298, 273, and 263 K) for two feed C_2_H_6_/C_2_H_4_ ratios, namely, 50/50 (*v/v*) and 1/15 (*v/v*), the latter representing the industrial feed composition after naphtha cracking (Figure 3e,f). It is seen that for the 50/50 mixture AC-F-15 exhibited a high C_2_H_6_/C_2_H_4_ selectivity of 5 at 10 mbar and 298 K, which then reached a steady value of 2.9 at 100 mbar and up to 1 bar. For the 1/15 mixture, the C_2_H_6_/C_2_H_4_ selectivity increased to 4 at 298 K and 1 bar, which is significantly higher than most of the C_2_H_6_-selective adsorbents at the same *P*, *T* conditions, such as C-CTS-2 (1.75),^54^ C-700-3 (3.2),^55^ Zn-ATA (1.8),^56^ and ZJU-HOF-1 (2.25),^57^ as depicted in the comparison plot of Figure 2f. At the lower temperatures tested, i.e., 273 and 263 K, for the 1/15 mixture, the C_2_H_6_/C_2_H_4_ selectivity at 1 bar reduced to 2.38 and 2.54, respectively.

**Figure 4:**
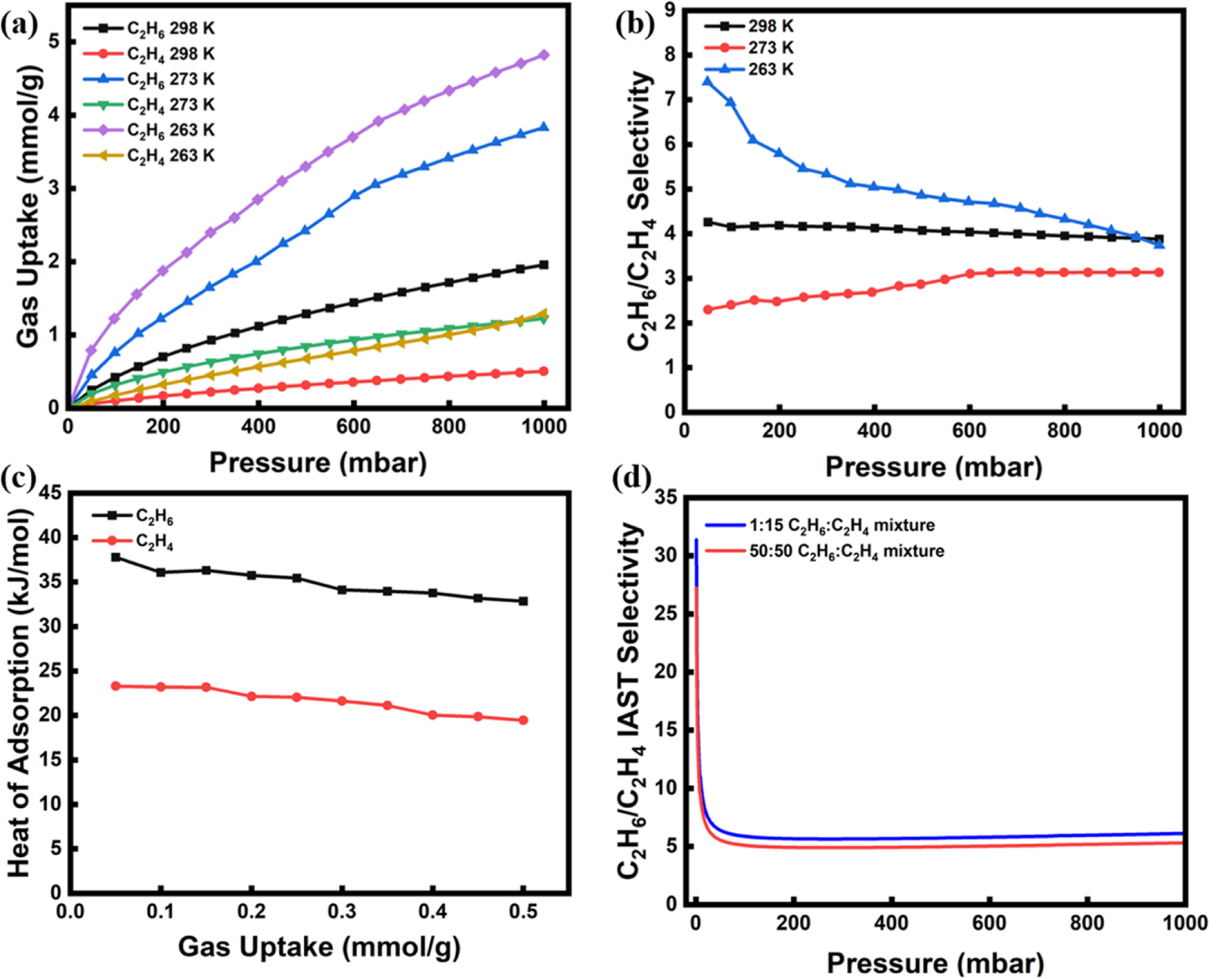
(a) C_2_H_6_ and C_2_H_4_ adsorption isotherms and (b) C_2_H_6_/C_2_H_4_ experimental selectivity at three different temperatures (298, 273, and 263 K), (c) isosteric heat of adsorption, and (d) C_2_H_6_/C_2_H_4_ IAST selectivity at 298 K for AC-F-20.

Given that in the separation of industrial C_2_H_6_/C_2_H_4_ streams selectivity may play a more crucial role than the capacity toward meeting the standards of C_2_H_4_ purity, the adsorbent with the highest selectivity (AC-F-20), as shown in Figure 2e, was also further studied. The adsorption performance of this adsorbent was assessed by adsorption experiments at different temperatures, i.e., 298, 273, and 263 K (Figure 4a), revealing that both C_2_H_6_ and C_2_H_4_ uptakes increased with a decrease in temperature. Indeed, for C_2_H_6_, the uptake increased from 1.96 at 298 K to 3.83 and 4.82 mmol/g at 273 and 263 K, respectively, at 1 bar; yet the respective increment in C_2_H_4_ uptake was only from 0.5 to 1.3 mmol/g. As a result, as shown in Figure 4b, AC-F-20 exhibited an outstanding selectivity of 7.4 at 50 mbar and 263 K, while at higher pressures, the selectivity reached a steady value of 4, which is almost the same as the selectivity at 298 K at this pressure. The adsorption sites that are based on van der Waal’s interactions are less specific sites typically allowing for multilayer physical adsorption, whereas fluorine-doped sites are more specific adsorption sites. Thus, at low pressure, the adsorption on the latter was dominant yielding higher selectivity, while at higher pressure, adsorption on the former sites became more dominant.

The isosteric heat of adsorption profiles for C_2_H_6_ and C_2_H_4_ on AC-F-20 are shown in Figure 4c. A stronger binding for C_2_H_6_ was evident throughout the uptake range examined. Indeed, at a loading of 0.5 mmol/g, the adsorption enthalpies for C_2_H_6_ and C_2_H_4_ were 33 and 19 kJ/mol, respectively, and the difference between the adsorption enthalpies of the two gases was almost constant throughout the examined gas loading range, signifying the constantly high paraffin affinity of the modified adsorbent and the homogeneous interactions of the surface with the C_2_H_6_ molecules. The IAST C_2_H_6_/C_2_H_4_ selectivity determined for two different feed mixtures is shown in Figure 4d. AC-F-20 exhibited a superior selectivity of 6.1 for a 1/15 mixture at 298 K and 1 bar, which was the highest under these conditions among all the fluorine-doped adsorbents developed here. At the lower pressure range, the selectivity reached a value of 10 at 10 mbar. The isosteric heat of adsorption also supported the two-sites theory.^58^ Adsorption on doped sites got isosteric heat values higher than pure physical adsorption; thus, as observed in Figure 4c, the isosteric heat decreased constantly when surface loading increased.

**Figure 5:**
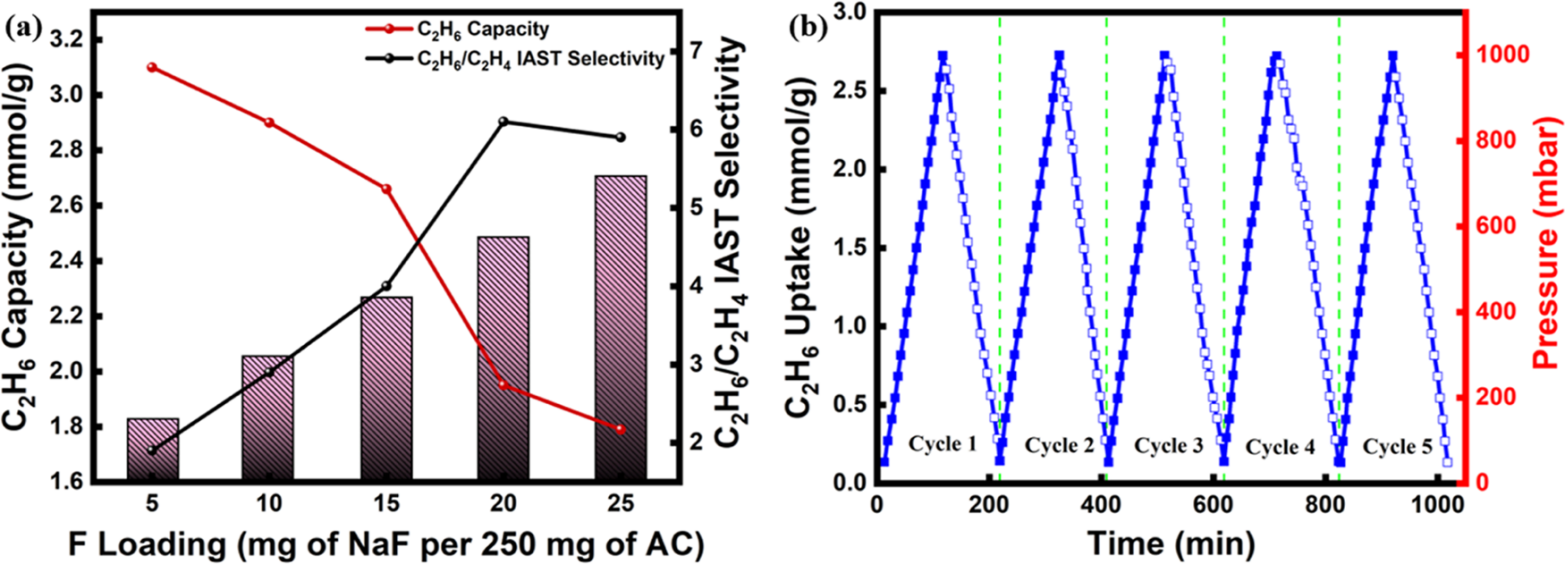
(a) Comparison of C_2_H_6_ capacity and C_2_H_6_/C_2_H_4_ IAST selectivity (1/15, *v/v*) as a function of the fluorine loading and (b) cyclic pressure-swing adsorption (PSA) adsorption–desorption performance of AC-F-15 at 298 K and up to 1 bar.

Figure 5a displays a performance comparison plot of all adsorbents developed in this work in terms of their C_2_H_6_ capacity and C_2_H_6_/C_2_H_4_ IAST selectivity for 1/15 (*v/v*) C_2_H_6_/C_2_H_4_ mixture at 1 bar and 298 K. The selectivity varied from 1.9 to 6.1 with an increase in fluorine loading, with a corresponding decrease in C_2_H_6_ capacity. For instance, the sample with the highest selectivity of 6.1 (AC-F-20) had a C_2_H_6_ capacity of 1.95 mmol/g. From this plot, the selection of the optimal adsorbent can be made according to the targets of an intended separation application. For example, AC-F-15 was found to maintain an optimum balance between selectivity and capacity, while AC-F-20 exhibits a significantly higher selectivity with a relatively lower capacity, and it should be preferred for applications where very high purity of the produced ethylene is required.

Adsorption–desorption cycles were conducted next to evaluate the cyclic performance of the adsorbents via pressure-swing adsorption (PSA). Five PSA cycles were performed between 1 bar and vacuum without any thermal treatment between the cycles. Figure 5b shows the cyclic performance of AC-F-15 at 298 K and up to 1 bar. Evidently, after five cycles, there was no decline in the capacity of the adsorbent, with a constant C_2_H_6_ uptake of 2.7 mmol/g. Notably, the adsorbed C_2_H_6_ was almost completely desorbed from the adsorbent by pressure swing alone, indicating that facile and energy-efficient regeneration can take place at ambient temperature, highlighting the potential of the developed functionalized adsorbents for industrial application.

### 3.3 Molecular Simulation Studies

**Figure 6:**
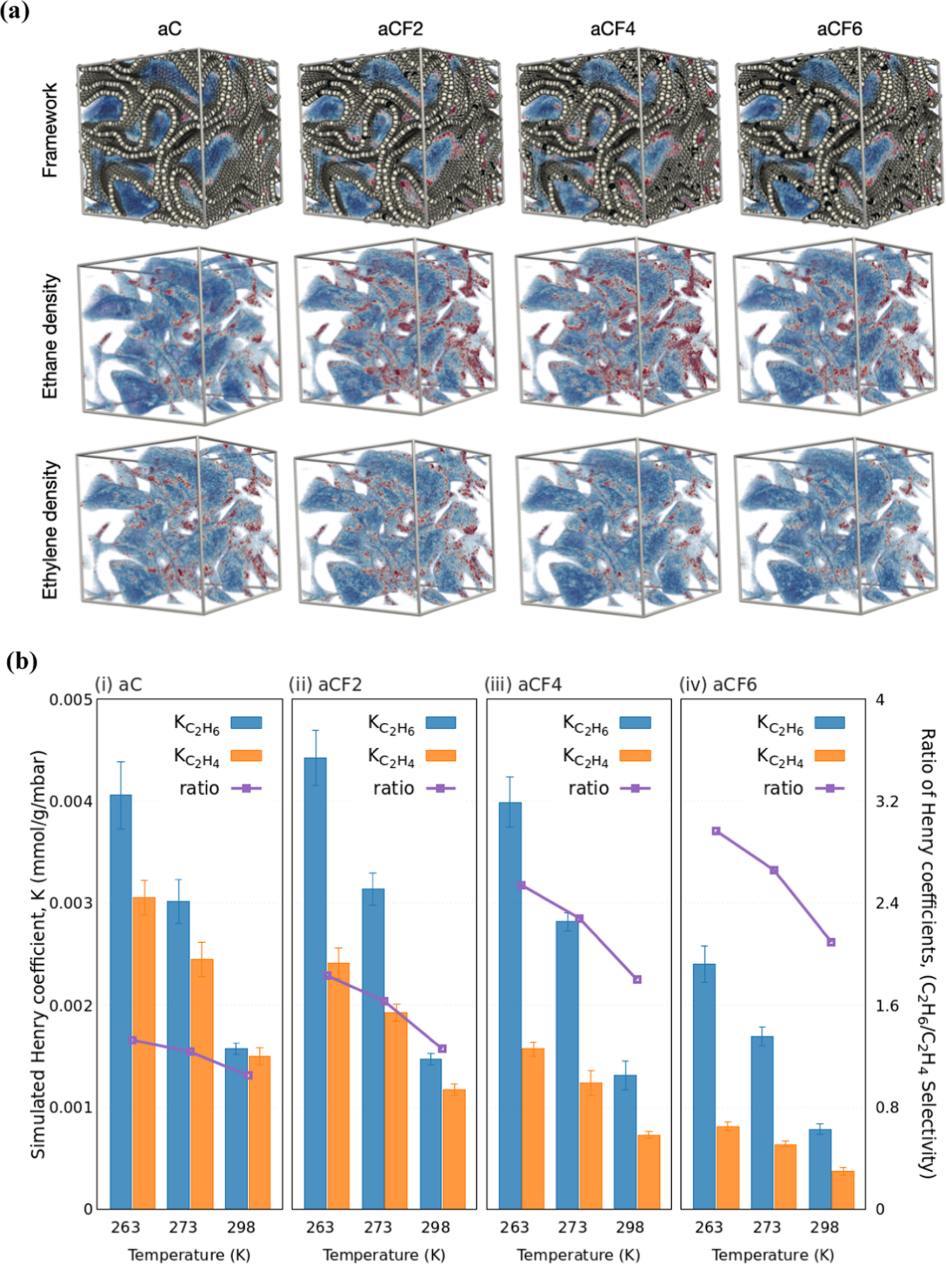
(a) Simulation models of activated carbon structures, including the all-carbon version (aC) and those decorated with 2% (aCF2), 4% (aCF4), and 6% (aCF6) fluorine atoms. The first row displays the frameworks of these samples, with carbon atoms represented by white spheres and fluorine atoms represented by black spheres, along with the density contours of adsorbed C_2_H_6_. The second and third rows illustrate the density contours of adsorbed C_2_H_6_ and C_2_H_4_, respectively, with the framework atoms omitted for the sake of clarity. In these contour plots, blue areas indicate regions of low adsorption density, while red areas represent regions of high density. (b) Simulated Henry coefficients for C_2_H_6_ and C_2_H_4_ adsorption on activated carbon structures, including (i) the all-carbon model (aC) and fluorine-doped models with (ii) 2% (aCF2), (iii) 4% (aCF4), and (iv) 6% (aCF6) fluorine atoms, at temperatures of 263, 273, and 298 K. The C_2_H_6_/C_2_H_4_ adsorption selectivities, represented by the ratio of the Henry coefficients for these gases, are plotted on the secondary *y*-axis in each panel.

Figure 6a shows the AC models, including the all-carbon structure and three fluorine-doped structures containing 2% (aCF2), 4% (aCF4), and 6% (aCF6) fluorine, along with the density contours of adsorbed ethane and ethylene at 298 K and 1 bar. The data are normalized to the highest observed density value, although the contour colors are still affected by the specific adsorbate loading within the pores. The density plots are “volume rendered,” with red indicating the denser regions of the adsorbed phase and blue representing less dense areas.

The adsorbed phases of both C_2_H_6_ and C_2_H_4_ are distributed throughout the accessible pore network of the samples, as depicted in Figure 6a. The simulation models reveal a nanoporous structure with continuous graphitized walls surrounding the pores. Both adsorbates display similar accessible porosity, with the pore size distribution presented in Figure S3, showing a pore limiting diameter of 5.08 Å and the largest cavity diameter of 20.85 Å. However, the adsorbed phases do not completely fill the pores; instead, they form a monolayer throughout the carbon walls, covering the surface area of the samples. The monolayer of adsorbed C_2_H_6_ is denser than that of C_2_H_4_, although neither phase is fully developed due to the low simulation pressure of 1 bar. It is evident that both gases occupy the same adsorption sites; however, C_2_H_6_ shows a slightly higher concentration in certain regions compared to C_2_H_4_. This difference is due to C_2_H_4_ being modeled as a rigid linear molecule, while C_2_H_6_, with its flexible bond oscillations, can fit more easily into confined spaces. As anticipated from the previously discussed experimental observations, the presence of fluorine on the carbon framework seems to increase the adsorption density for both gases with the effect being more pronounced for C_2_H_6_ than for C_2_H_4_.

These findings are further analyzed using the radial distribution functions (RDFs) of ethane and ethylene molecules confined within the pores of the fluorinated samples at 298 K and 1 bar, as shown in Figure S4 in the Supporting Information. The radii represent the center-of-mass (COM) distances between the fluorine atoms in the carbon frameworks and the CH_3_ units of ethane or the CH_2_ units of ethylene. The inset plots focus on the peak range of the RDF curves, showing that adsorbed ethane is located closer to the fluorine atoms (peak at approximately 7.1 Å) compared to that of ethylene (peak at approximately 7.8 Å). The RDF curves also indicate that the adsorbed layer of ethane is slightly denser than that of ethylene and that ethane molecules are adsorbed closer to the fluorine sites than to ethylene molecules. A series of snapshot configurations of ethane and ethylene adsorption in pure and fluorinated carbon frameworks with the fluorine contents of 0% (aC), 2% (aCF2), 4% (aCF4), and 6% (aCF6) at 298 K and 1 bar are also appended in Figure S5.

The ethane-selective performance of the fluorine-doped carbon samples is clearly illustrated by the differences in the simulated Henry adsorption coefficients (estimated using eq S4) shown in Figure 6b. These coefficients were calculated using the Widom insertion method for all four samples at three temperatures, i.e., 263, 273, and 298 K. For the pure carbon sample (aC) at 263 K, the Henry coefficient for C_2_H_6_ adsorption is 4.0 × 10^–3^ mmol/g/mbar, while for C_2_H_4_, it is 3.0 × 10^–3^ mmol/g/mbar. When the sample is doped with 2% fluorine (aCF2), the Henry coefficient for C_2_H_6_ adsorption increases to 4.4 × 10^–3^ mmol/g/mbar, while that for C_2_H_4_ decreases to 2.4 × 10^–3^ mmol/g/mbar. With a further increase in fluorine content to 4% (aCF4), the Henry coefficient for C_2_H_6_ adsorption decreases to 3.9 × 10^–3^ mmol/g/mbar, while for C_2_H_4_, it drops further to 1.5 × 10^–3^ mmol/g/mbar. It appears that the C_2_H_6_ density (and thus capacity) increases up to a certain level of fluorine loading, after which it begins to decrease (as seen in aCF4). This trend aligns with the experimental isotherms (Figure 2), where beyond a certain threshold of fluorine loading the capacity decreases. A possible explanation is that once the fluorine content surpasses a certain threshold, the increase in the framework’s mass outweighs the increase in excess adsorbate density, resulting in a reduction in overall adsorption capacity.

The ratio of the Henry adsorption coefficients is related to the adsorption selectivity at low loading, which is also plotted in Figure 6b. Specifically, the ratio of the Henry coefficients for C_2_H_6_ to C_2_H_4_ adsorption in the pure carbon sample is 1.32 at 263 K and drops to 1.05 at 298 K. Similarly, for the carbon sample with 6% fluorine content, the ratio is 2.54 at 263 K and 1.8 at 298 K. The overall trend from the molecular simulations shows that C_2_H_6_/C_2_H_4_ selectivity decreases with increasing temperature and increases with higher fluorine content. This trend is in agreement with the selectivities derived from the experimental adsorption isotherms of C_2_H_6_ and C_2_H_4_ on pure carbon and fluorine-doped carbon samples, as shown in Figures 2 and 3, respectively. The simulated adsorption enthalpies are listed in Table 3. It is evident that C_2_H_6_ exhibits stronger adsorption on the samples compared to C_2_H_4_, and the adsorption enthalpy increases with higher fluorine content in the framework, in agreement with the experimental findings.

**Table 3:** Simulated Adsorption Enthalpies of C_2_H_6_ and C_2_H_4_ on Pure Carbon (aC) and Fluorine-Doped Carbon Models (aCF2, aCF4, and aCF6) at Zero Coverage

	sample	aC	aCF2	aCF4	aCF6
Δ*H* (kJ/mol)	ethane	17.61	20.52	20.69	20.84
	ethylene	13.25	13.48	14.28	14.35

### 3.4 Kinetic Analysis

**Figure 7:**
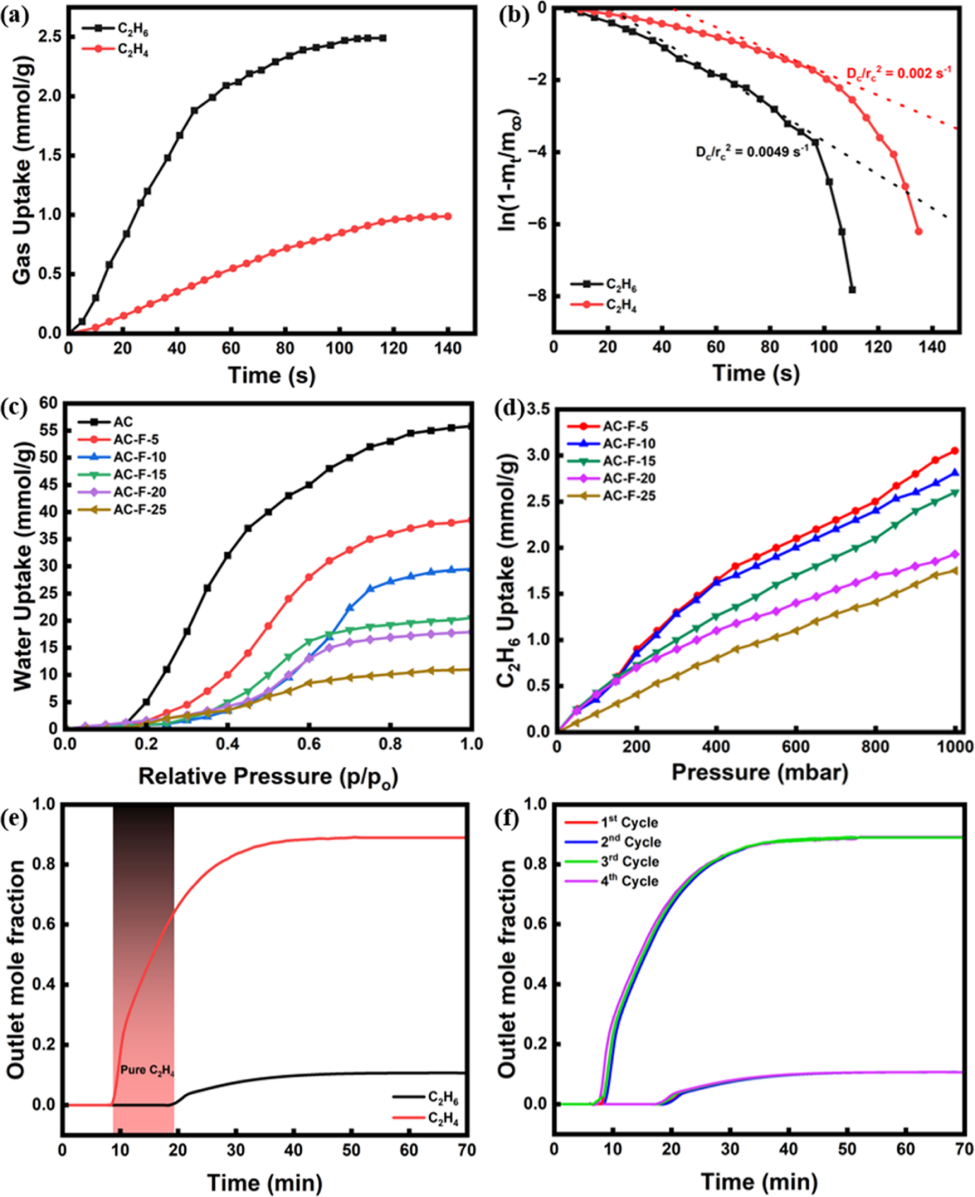
(a) Dynamic uptake of C_2_H_6_ and C_2_H_4_ at 298 K and 1 bar and (b) kinetic curves for the estimation of diffusion parameters for AC-F-15. (c) Water vapor adsorption isotherms and (d) C_2_H_6_ adsorption isotherms of moisture-exposed AC-F samples at 298 K and up to 1 bar. (e) Breakthrough adsorption curves for 1/9 (*v/v*) C_2_H_6_/C_2_H_4_ mixture at ambient temperature and (f) cyclic performance for up to 4 breakthrough cycles for AC-F-15.

Kinetic analysis of adsorption processes is necessary to acquire insights into the mass transfer constraints involved and the mechanism of adsorption. Accordingly, dynamic adsorption experiments were conducted with the AC-F-15 adsorbent at 298 K and 1 bar, and the corresponding time-dependent uptake curves are shown in Figure 7a. As observed, C_2_H_6_ exhibits a higher adsorption rate, and it reached saturation faster than C_2_H_4_, suggesting faster diffusion and binding of the paraffin molecules. In these dynamic experiments, C_2_H_6_ reached a saturation uptake of 2.5 mmol/g at 1 bar which is in close agreement with the equilibrium value obtained (2.7 mmol/g). At the beginning of the adsorption, the uptake rate is faster due to the availability of a high number of easily accessible adsorption sites as well as due to the fact that adsorption and diffusion take place initially under isothermal conditions. As adsorption progresses, the local temperature increases slightly due to the exothermic nature of adsorption causing, in conjunction with the decrease in the number of available adsorption sites, a reduction in physisorption rate with time. C_2_H_4_ took longer time than C_2_H_6_ to reach equilibrium, implying a lower affinity and higher mass transfer resistance. To quantify the pore diffusion characteristics, a micropore diffusional model^59^ (eq S6) was used. The slope of the tangent ( value within the range of 40–80%) of the curve  vs *t*, as shown in Figure 7b, was approximated, from which the apparent diffusional time constants (*D*_c_/*r*_c_^2^) for both gases were estimated as 0.0049 and 0.002 s^–1^ for C_2_H_6_ and C_2_H_4_, respectively, confirming that C_2_H_4_ moves slower than C_2_H_6_ inside the pores due to higher surface barrier. Accordingly, the kinetic selectivity of AC-F-15 adsorbent was 2.45, as calculated by the ratio of the time constants of the two gases, and is higher than that of reported state of the art adsorbents, such as boron nitride (2.39),^59^ ZIF-7 (2.37),^60^ and zeolite 13X (1.5).^61^

### 3.5 Moisture Stability

The industrial feed after naphtha cracking generally contains traces of moisture, which can compromise the performance of adsorbents and reduce their lifetime. An ideal absorbent should have a low moisture uptake along with good structural stability when exposed to moisture. To explore this in our adsorbents, water vapor sorption experiments were carried out at 298 K and up to 100% relative humidity (RH). In addition, the moisture-exposed samples were subsequently tested for C_2_H_6_ adsorption. The water vapor isotherms in Figure 7c show a systematic suppression of the hydrophilicity of the adsorbents with an increase in fluorine doping. Indeed, pure AC had the highest water uptake (maximum of 56 mmol/g), which gradually decreased to 11 mmol/g for AC-F-25 at the same RH, i.e., undergoing a 5-fold reduction in water uptake. This drop in moisture uptake is corroborated by the degree of functionalization and the reduction in hydrophilicity and surface area, as confirmed above by the porosimetry studies. Fluorine is well-known for its hydrophobicity when bonded to carbon due to the fact that its strong electronegativity can draw the shared electrons and reduce the polarity of the neighboring atoms and subsequently weaken the interaction between the carbon surface and water, resulting in reduced wettability.^62^ The moisture-exposed fluorine-doped samples were tested for C_2_H_6_ adsorption at 298 K. The obtained C_2_H_6_ adsorption isotherms of the prehumidified adsorbents are shown in Figure 7d. A minor decrease in C_2_H_6_ uptake was observed compared to the dry samples, i.e., by 1.6, 4, 2.2, 1.3, and 2.6% for AC-F-5, AC-F-10, AC-F-15, AC-F-20, and AC-F-25, respectively, indicating that the C_2_H_6_ adsorption capacity was not significantly affected by the exposure to moisture.

### 3.6 Breakthrough Studies

Breakthrough experiments on AC-F-15 were carried out at ambient temperature in a fixed bed with dimensions of 15 cm × 1 cm using a 1/9 (*v/v*) C_2_H_6_/C_2_H_4_ mixture as the feed. Cyclic breakthrough experiments were also conducted to evaluate the sustained separation performance of the adsorbent. Based on the breakthrough curves (Figure 7e), C_2_H_4_ was eluted out of the bed after 7 min while C_2_H_6_ breaks through at 18 min, i.e., with a time difference of 11 min between the two gases, thus indicating the selective adsorption of C_2_H_6_ enabling sufficient processing time for the continuous production of polymer-grade C_2_H_4_. This breakthrough time gap between the two gases is higher than some of the best-performing C_2_H_6_-selective adsorbents reported in the literature at similar conditions, such as PCN-245 (2 min),^18^ Zr-bptc (3 min),^63^ and MUF-15 (7 min).^64^ Productivity of C_2_H_4_ was estimated to be 1.6 mmol/g, which is higher than the reported C_2_H_4_ productivities by top-performing adsorbents such as BUT-10 (0.12 mmol/g),^65^ Fe_2_(O)_2_(dobdc) (0.79 mmol/g),^66^ and MAF-49 (0.28 mmol/g).^67^ Furthermore, the C_2_H_6_ productivity, as calculated based on the saturation points of the breakthrough curves, was found to be 0.47 mmol/g, yielding a breakthrough selectivity of 2.64, which is higher than the selectivity of other reported highly performing ethane-selective adsorbents such as COF-1 (1.44),^68^ UPC-612 (0.56),^69^ and 75CPDA@A-AC (1.78).^70^ Multicycle breakthrough experiments were also carried out without any thermal regeneration of the adsorbent bed in between the cycles, as after every cycle helium was only purged for 20 min at 298 K at 1 bar for regenerating the bed. These cyclic experiments showed that the time difference between the elution of C_2_H_6_ and C_2_H_4_ remained almost unchanged for the four cycles tested, confirming the cyclable performance of the adsorbent. The results of these experiments demonstrate that the developed C_2_H_6_-selective adsorbents can produce continuously polymer-grade C_2_H_4_ of purity on the order of 99.99% (according to the detection capability of the used DSMS) through a cyclic adsorption process without involvement of thermal regeneration.

## 4 Conclusions

Fluorine-functionalized ethane-selective adsorbents of superior performance and stability were developed based on commercial activated carbon, an abundant and cost-effective material. Specific interactions between the surface-doping fluorine atoms and ethane molecules and porosity modulation resulted in enhanced ethane selectivity. Indeed, modification with fluorine brought about a remarkable ethane/ethylene equilibrium selectivity of up to 3.9 (for AC-F-20) at 298 K and 1 bar. The ethane selectivity observed in the fluorine-doped carbons was further illustrated through Monte Carlo simulations and density contours of the adsorbed components at 298 K and 1 bar, comparing a simulated all-carbon model with three fluorine-doped carbon models of varying fluorine content in the framework. Moreover, the simulated Henry adsorption coefficients agreed with the experimental findings, showing that ethane selectivity generally increases with a higher fluorine content in the sample and decreases with increasing temperature. Mixed-gas (C_2_H_6_:C_2_H_4_, 1:9 *v/v*) breakthrough experiments revealed a productivity of 1.6 mmol/g of polymer-grade ethylene by a cyclic adsorption separation process under ambient temperature. The modified adsorbents exhibited also enhanced hydrophobicity, thus limiting competitive adsorption with water and enhancing stability in the presence of moisture, while their C_2_H_6_ capacity was not compromised by the presence of humidity. Multicycle equilibrium and breakthrough experiments without thermal regeneration confirmed the cyclability and regeneration of the adsorbents for continuous industrial operation. Future research should focus on the optimization of fluorine-doping levels and of the functionalization strategies to further enhance selectivity and stability. Life cycle assessment and technoeconomic analysis of these modified adsorbents is also crucial for assessing their economic viability and industrial potential. Additionally, in-depth exploration of the underlying molecular mechanisms of fluorine-ethylene and fluorine-ethane interactions using advanced computational techniques could provide new insights toward optimal design of the novel adsorbents.

## Supporting Information Available

Amount of NaF used per 250 mg of AC in the preparation of the functionalized AC samples (Table S1); (a) micropore and (b) mesopore size distribution of pristine and fluorine-modified activated carbon samples (Figure S1); SEM images of (a) AC, (b) AC-F-5, (c) AC-F-10, (d) AC-F-15, (e) AC-F-20, and (f) AC-F-25 (Figure S2); fitting parameters of the DSL model for C_2_H_6_ and C_2_H_4_ adsorption on AC and AC-F-15 (Table S2); C_2_H_6_/C_2_H_4_ separation performance of C_2_H_6_-selective adsorbents from the literature (Table S3); and pore size distribution (PSD) of the simulated AC model (Figure S3); radial distribution functions (RDFs) for ethane and ethylene adsorption in fluorinated carbon sample models (aCF2, aCF4, and aCF6) at 298 K and 1 bar (Figure S4); snapshot configurations of ethane and ethylene adsorption in pure and fluorinated carbon frameworks with fluorine contents of 0% (aC), 2% (aCF2), 4% (aCF4), and 6% (aCF6) at 298 K and 1 bar (Figure S5) (PDF)
