# Supplementary material for: High-Purity Ethylene Production from Ethane/Ethylene Mixtures at Ambient Conditions by Ethane-Selective Fluorine-Doped Activated Carbon Adsorbents
Source: ACS Appl Mater Interfaces. 2025 Jan 25;17(5):8619–33. doi: 10.1021/acsami.4c20772 (PMC11803616; doi:10.1021/acsami.4c20772)
Supplement: Supplementary file 1 — am4c20772_si_001.pdf [file am4c20772_si_001.pdf]

Supporting Information

High-purity ethylene production from ethane/ethylene mixtures at ambient conditions by ethane-selective fluorine-doped activated carbon adsorbents

Fahmi Anwar[1,2,3], Anish Mathai Varghese[1,2], Suresh Kuppireddy[3], Anastasios Gotzias[4], Maryam Khaleel[1,5], Kean Wang[6], Georgios N. Karanikolos[7]*

[1]Department of Chemical and Petroleum Engineering, Khalifa University, P.O. Box 127788, Abu Dhabi, UAE

[2]Center for Catalysis and Separation (CeCaS), Khalifa University, P.O Box 127788, Abu Dhabi, UAE

[3]Renewable and Sustainable Energy Research Center, Technology Innovation Institute (TII), P.O. Box 9639, Masdar City, Abu Dhabi, UAE

[4]Institute of Nanoscience and Nanotechnology, National Center for Scientific Research Demokritos, Athens 15310, Greece

[5]Research and Innovation Center for CO_2 and H_2 (RICH), Khalifa University, P.O. Box 127788, Abu Dhabi, UAE

[6]Food, Chemical and BioTechnology Cluster, Singapore Institute of Technology, 10 Dover Drive, Singapore 138683

[7]Department of Chemical Engineering, University of Patras, 26504 Patras, Greece

*Corresponding author email: karanikolos@chemeng.upatras.gr

Table S1: Amount of NaF used per 250 mg of AC in the preparation of the functionalized AC samples.

Sample Code	Amount of NaF (mg)
AC	-
AC-F-5	5
AC-F-10	10
AC-F-15	15
AC-F-20	20
AC-F-25	25

S1. Characterization

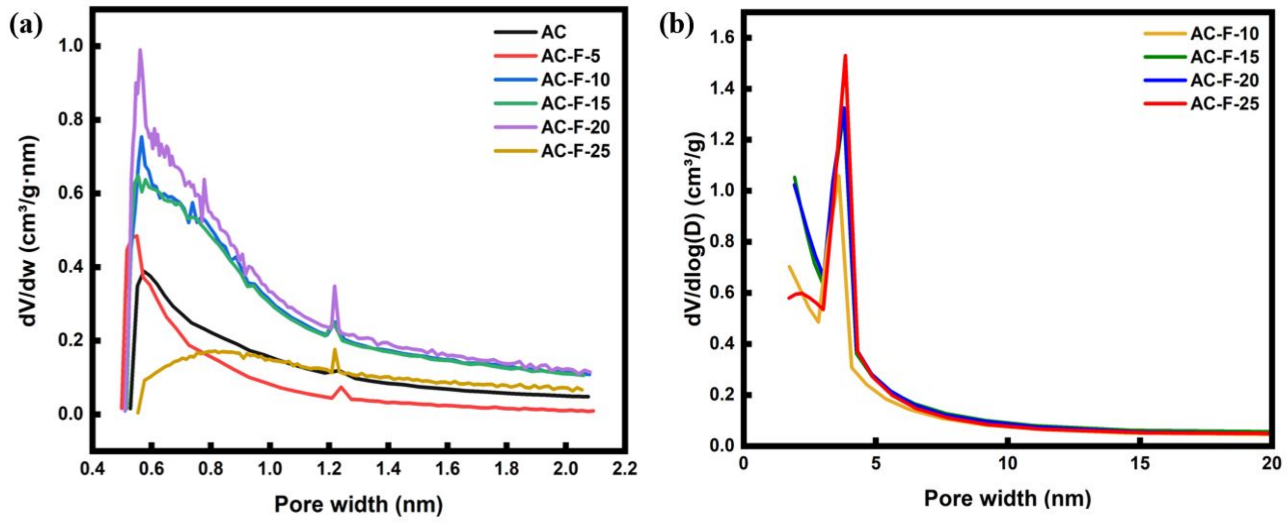

Figure S1: (a) Micropore and (b) mesopore size distribution of pristine and fluorine-modified activated carbon samples.

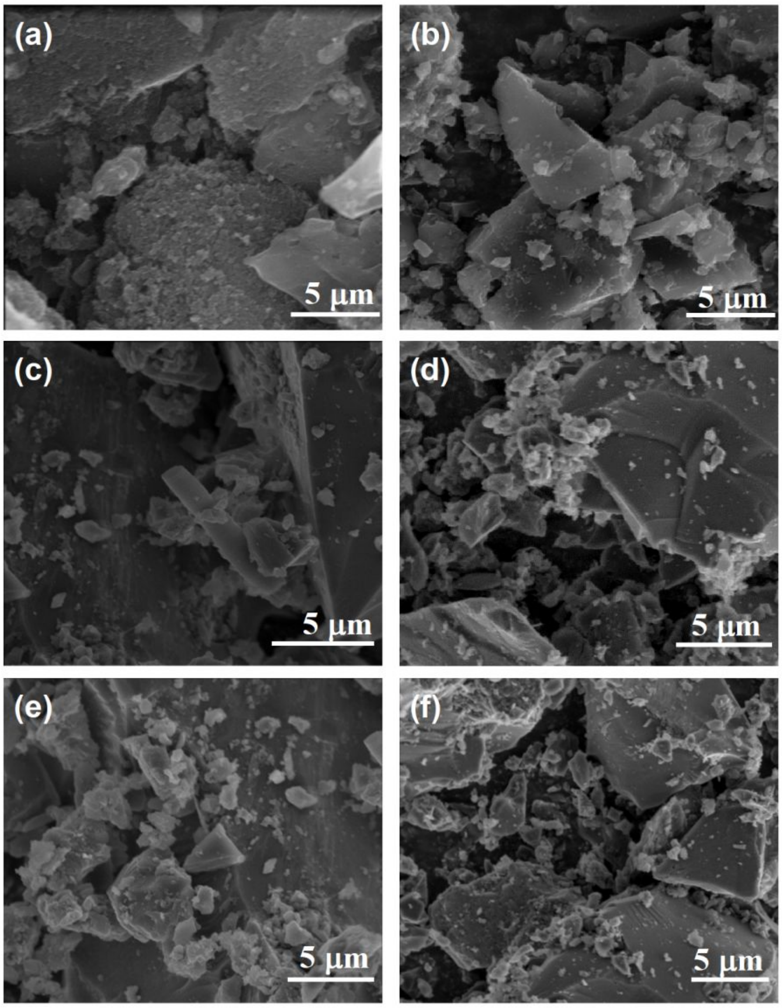

Figure S2: SEM images of (a) AC, (b) AC-F-5, (c) AC-F-10, (d) AC-F-15, (e) AC-F-20, and (f) AC-F-25.

S2. Equations and Models

The adsorption isotherms at different temperatures were fitted using the Dual-Site Langmuir (DSL) model [1] which is expressed below:

$$q = \frac{q_{s1} b_1 p}{1 + b_1 p} + \frac{q_{s2} b_2 p}{1 + b_2 p} \quad \text{(S1)}$$

where q is the equilibrium adsorption uptake, p is the gas pressure, and q_{s1}-q_{s2} and b_1-b_2 are the saturation capacities and affinity coefficients of sites 1 and 2, respectively.

The experimental isotherms were fitted with the DSL model with $R^2 > 0.999$ (fitting parameters shown in Table S2), implying a good agreement between the chosen model and the experimental values. After fitting, for the estimation of Q_{st}, the Clausius-Clayperon equation [2] was used, as given below:

$$\ln p = -\frac{\Delta H_S}{RT} + C \quad \text{(S2)}$$

where p is the pressure (bar), ΔH_s is the adsorption enthalpy (J/mol), R is the universal gas constant (J/mol.K), T is the temperature, and C is the integration constant.

Table S2: Fitting parameters of the DSL model for C_2H_6 and C_2H_4 adsorption on AC and AC-F-15.

Sample	T (K)	q_{s1} (mmol/g)		q_{s2} (mmol/g)		b_1 (bar^{-1})		b_2 (bar^{-1})	
		C_2H_6	C_2H_4	C_2H_6	C_2H_4	C_2H_6	C_2H_4	C_2H_6	C_2H_4
AC	298	5.37	4.71	2.4	2.22	0.001	0.012	0.002	0.002
	273	5.74	4.82	2.16	2.67	0.002	0.003	0.003	0.003
	263	7.11	5.38	1.45	3.1	0.02	0.001	0.001	0.002
AC-F-15	298	6.12	3.42	1.91	2.33	0.005	0.003	0.002	0.001
	273	6.25	3.68	1.21	1.52	0.004	0.002	0.003	0.001
	263	6.81	4.05	1.08	1.18	0.001	0.001	0.001	0.001

To predict the binary gas adsorption behavior, the ideal adsorbed solution theory (IAST) developed by Myers and Prausnitz [3] was used to estimate the selectivity of the adsorbents for different feed gas concentrations. The selectivity is given as:

$$S = \frac{x_1/x_2}{y_1/y_2} \quad \text{(S3)}$$

where x_i and y_i are the mole fractions of component i ($i = 1$ for C_2H_6 and $i = 2$ for C_2H_4) in the adsorbed and bulk gas phases, respectively.

For the Monte Carlo Simulations, the pressure for a given uptake q for the three adsorption isotherms at temperatures T_1, T_2, and T_3, with corresponding Henry coefficients K_1, K_2, and K_3, is expressed as

$$p(q,T_i) = {}^{q}/_{K_i} \tag{S4}$$

where i = 1, 2, 3 refers to the corresponding temperature T_i. By plotting $\ln(p(q,T_i))$ as a function of the inverse temperature $1/T_i$ a straight line is obtained. The slope of this line, S, is related to the isosteric heat of adsorption through the equation

$$^{\Delta H}/_{R} = S \tag{S5}$$

where R is the universal gas constant [4]. The pore size distribution (PSD) of the simulated AC model was computed over 10,000 cycles, with the PSD being output every 100 cycles. The probe distance between the framework atoms was defined by their σ diameter of the Lennard-Jones potential.

To quantify the pore diffusion characteristics, the following kinetic micro-pore model [5] was used:

$$1 - \frac{m_t}{m_\infty} = \frac{6}{\pi^2} exp\left(\frac{-\pi^2 D_C t}{r_c^2}\right) \tag{S6}$$

where m_t and m_∞ refer to the amount of gas adsorbed (m_g) at time t and at saturation respectively, r_c refers to the intracrystalline radius, and D_c represents the intracrystalline diffusivity. Accordingly, the kinetic selectivity of the adsorbent was calculated using Eq. S7, [6] given below:

$$S_k = \frac{\left(D_C/r_C^2\right)_i}{\left(D_C/r_C^2\right)_j} \tag{S7}$$

where the numerator and denominator accommodate the faster and slower diffusing components, respectively.

The breakthrough productivity (q_i) was determined by the below equation [7]:

$$q_i = \frac{C_i p}{m_{ads} RT}\left(V.t - V_{col} + \frac{m_{ads}}{\rho_{ads}}\right) \quad \text{(S8)}$$

where C_i is the initial concentration of component i, m_{ads} is the amount of adsorbent loaded in the column, p is the pressure inside the column, V is the total gas flowrate, V_{col} is the volume of the adsorbent bed, ρ_{ads} is the skeletal density of the adsorbent, and t is the dynamic adsorption time as calculated by Eq. S9 below:

$$t = \int_0^t \left(1 - \frac{C_t}{C_i}\right) dt \quad \text{(S9)}$$

where C_t is the outlet gas concentration of component i at time t.[8] The breakthrough selectivity is given by

$$S = \frac{q_1/x_1}{q_2/x_2} \quad \text{(S10)}$$

where q_i and x_i are the productivity and mole fraction of component i (i = 1 for C_2H_6 and i = 2 for C_2H_4) respectively.

Table S3: C_2H_6/C_2H_4 separation performance of C_2H_6-selective adsorbents from literature.

Adsorbent	C_2H_6/C_2H_4 ratio	IAST C_2H_6/C_2H_4 Selectivity	C_2H_6 Capacity (*mmol/g*)	Conditions	Ref
AC-F-15	1/15	4	2.66	298 K and 1 bar	This work
AC-F-20	1/15	6.1	1.95	298 K and 1 bar	This work
AC-F-25	1/15	5.9	1.79	298 K and 1 bar	This work
NUM-9	1/9	1.63	2.1	313 K and 1 bar	[9]
MIL-142A	1/1	1.5	3.8	298 K and 1 bar	[10]
MIL-53-FA	1/15	1.9	3.7	308 K and 1 bar	[11]
PCN-245	1/1	1.6	3.27	298 K and 1 bar	[12]

MUF-15	1/1	1.95	4.7	293 K and 1 bar	13
Zn-PNMI	1/15	1.42	1.61	298 K and 1 bar	14
Cd-PNMI	1/15	1.27	1.84	298 K and 1 bar	14
Sm-BTC	1/15	1.7	1.65	298 K and 1 bar	15
Y-BTC	1/15	1.9	3.44	298 K and 1 bar	15
UiO-66-2CF_3	1/1	2.5	1.8	298 K and 10 bar	16
FMOF-2	1/1	3.3	1.2	303 K and 0.4 bar	17
LIFM-61	1/1	1.32	1.7	298 K and 1 bar	18
Ni(IN)$_2$	1/1	2.45	3	298 K and 1 bar	19
Zn-ATA	1/9	1.8	1.2	298 K and 1 bar	20
Dia-4-Ni	1/9	1.76	4.46	298 K and 1 bar	21
MOF-545	1/15	1.31	3.2	298 K and 1 bar	22

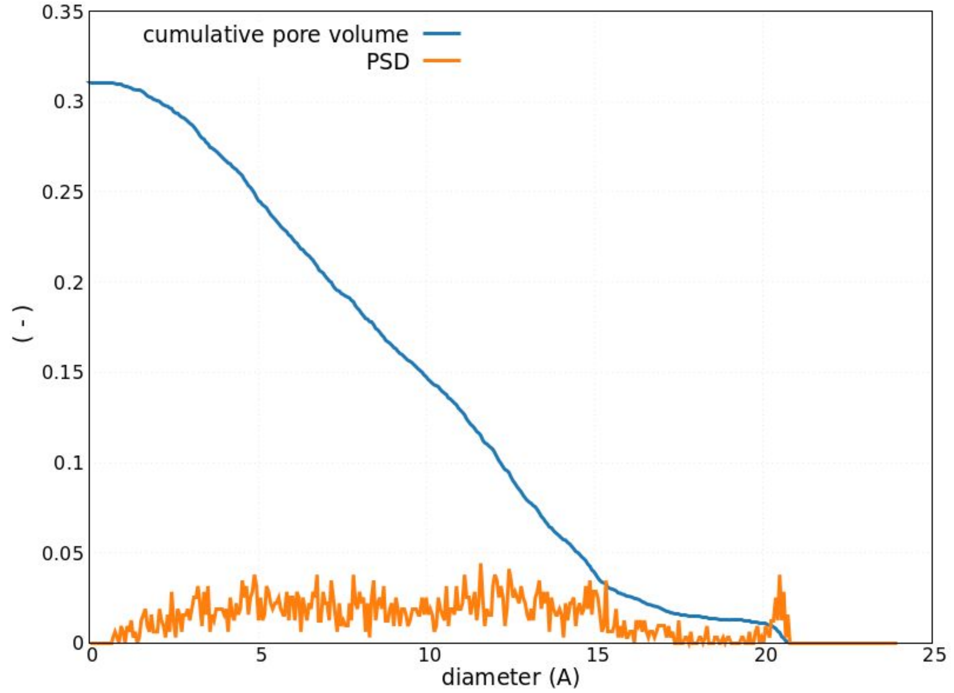

Figure S3: Pore size distribution (PSD) of the simulated AC model. The cumulative pore volume value at d=0 is related to the Helium void fraction listed in Table 1.

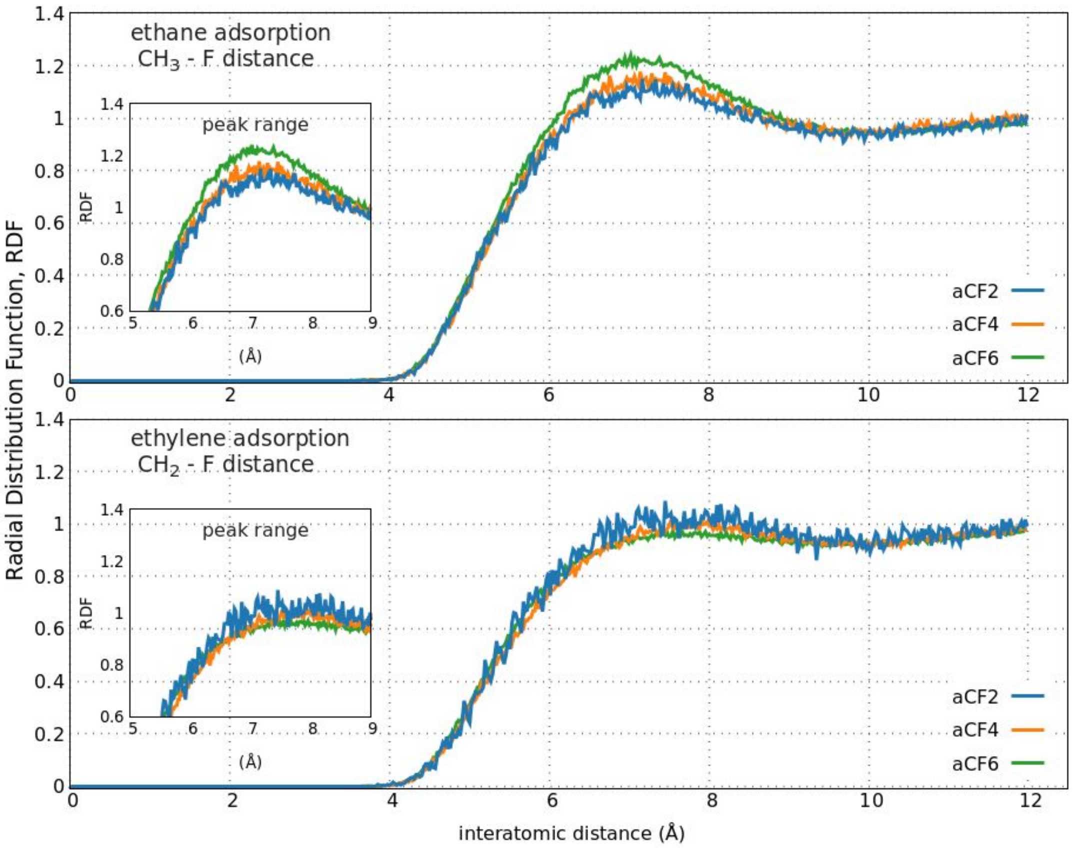

Figure S4: Radial distribution functions (RDFs) for ethane (top) and ethylene (bottom) adsorption in fluorinated carbon sample models (aCF2, aCF4, and aCF6) at 298 K and 1 bar. The radii represent the center-of-mass (COM) distances between the fluorine (F) atoms in the carbon frameworks and the CH_3 units of ethane or the CH_2 units of ethylene. The inset plots focus on the peak range of the RDF curves, showing that adsorbed ethane is located closer to the fluorine atoms (peak at approximately 7.1 Å) compared to ethylene (peak at approximately 7.8 Å). Moreover, the adsorbed layer density of ethane increases with higher fluorine content in the framework, while the adsorption density of ethylene decreases as the fluorine content increases.

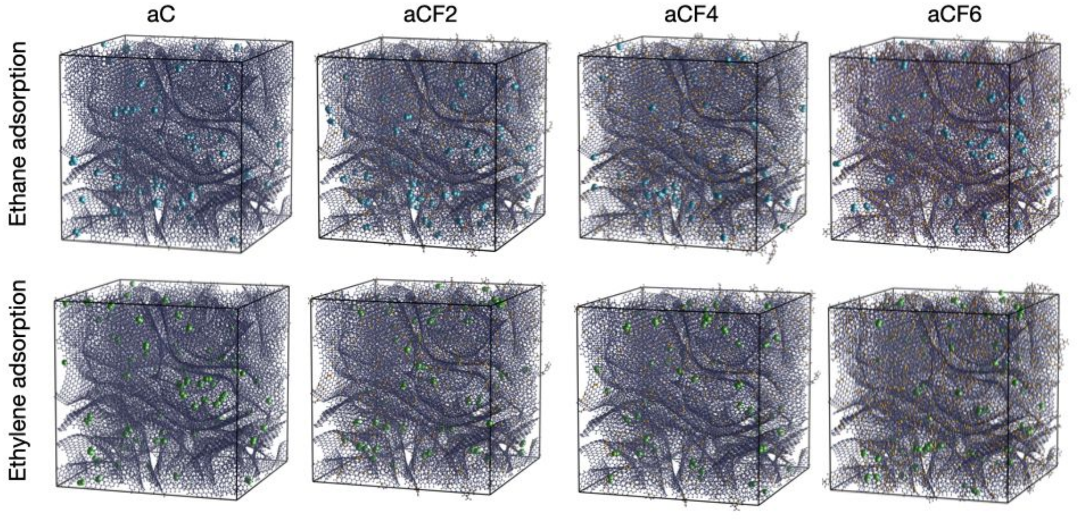

Figure S5: Snapshot configurations of ethane (top) and ethylene (bottom) adsorption in pure and fluorinated carbon frameworks with fluorine contents of 0% (aC), 2% (aCF2), 4% (aCF4), and 6% (aCF6) at 298 K and 1 bar. Carbon atoms are depicted in dark blue, fluorine atoms in brown, ethane molecules in cyan, and ethylene molecules in green.

References

(1) Xiang, H.; Shao, Y.; Ameen, A.; Chen, H.; Yang, W.; Gorgojo, P.; Siperstein, F. R.; Fan, X.; Pan, Q. Adsorptive Separation of C_2H_6/C_2H_4 on Metal-Organic Frameworks (MOFs) with Pillared-Layer Structures. *Sep. Purif. Technol.* **2020**, *242*, 116819.

(2) Varghese, A. M.; Reddy, K. S. K.; Bhoria, N.; Singh, S.; Pokhrel, J.; Karanikolos, G. N. Enhancing Effect of UV Activation of Graphene Oxide on Carbon Capture Performance of Metal-Organic Framework / Graphene Oxide Hybrid Adsorbents. *Chem. Eng. J.* **2021**, *420*, 129677.

(3) Ma, C.; Wang, X.; Wang, X.; Yuan, B.; Wu, Y.; Li, Z. Novel Glucose-Based Adsorbents (Glc-As) with Preferential Adsorption of Ethane over Ethylene and High Capacity.

Chem. Eng. Sci. **2017**, *172*, 612–621.

(4) Gotzias, A.; Kouvelos, E.; Sapalidis, A. Computing the Temperature Dependence of Adsorption Selectivity in Porous Solids. *Surf. Coatings Technol.* **2018**, *350*, 95–100.

(5) Saha, D.; Orkoulas, G.; Yohannan, S.; Ho, H. C.; Cakmak, E.; Chen, J.; Ozcan, S. Nanoporous Boron Nitride as Exceptionally Thermally Stable Adsorbent: Role in Efficient Separation of Light Hydrocarbons. *ACS Appl. Mater. Interfaces* **2017**, *9*, 14506–14517.

(6) Chung, K.; Park, D.; Kim, K. M.; Lee, C. H. Adsorption Equilibria and Kinetics of Ethane and Ethylene on Zeolite 13X Pellets. *Microporous Mesoporous Mater.* **2022**, *343*, 112199.

(7) Liang, W.; Zhang, Y.; Wang, X.; Wu, Y.; Zhou, X.; Xiao, J.; Li, Y.; Wang, H.; Li, Z. Asphalt-Derived High Surface Area Activated Porous Carbons for the Effective Adsorption Separation of Ethane and Ethylene. *Chem. Eng. Sci.* **2017**, *162*, 192–202.

(8) Liang, W.; Wu, Y.; Xiao, H.; Xiao, J.; Li, Y.; Li, Z. Ethane-Selective Carbon Composites CPDA@A-ACs with High Uptake and Its Enhanced Ethane/Ethylene Adsorption Selectivity. *AIChE J.* **2018**, *64*, 3390–3399.

(9) Yang, S. Q.; Sun, F. Z.; Liu, P.; Li, L.; Krishna, R.; Zhang, Y. H.; Li, Q.; Zhou, L.; Hu, T. L. Efficient Purification of Ethylene from C2 Hydrocarbons with an C_2H_6/C_2H_2-Selective Metal-Organic Framework. *ACS Appl. Mater. Interfaces* **2021**, *13*, 962–969.

(10) Chen, Y.; Wu, H.; Lv, D.; Shi, R.; Chen, Y.; Xia, Q.; Li, Z. Highly Adsorptive Separation of Ethane/Ethylene by An Ethane-Selective MOF MIL-142A. *Ind. Eng. Chem. Res.* **2018**, *57*, 4063–4069.

(11) Peng, J.; Sun, Y.; Wu, Y.; Lv, Z.; Li, Z. Selectively Trapping Ethane from Ethylene on

Metal-Organic Framework MIL-53(Al)-FA. *Ind. Eng. Chem. Res.* **2019**, *58*, 8290–8295.

(12) Lv, D.; Shi, R.; Chen, Y.; Wu, Y.; Wu, H.; Xi, H.; Xia, Q.; Li, Z. Selective Adsorption of Ethane over Ethylene in PCN-245: Impacts of Interpenetrated Adsorbent. *ACS Appl. Mater. Interfaces* **2018**, *10*, 8366–8373.

(13) Qazvini, O. T.; Babarao, R.; Shi, Z. L.; Zhang, Y. B.; Telfer, S. G. A Robust Ethane-Trapping Metal-Organic Framework with a High Capacity for Ethylene Purification. *J. Am. Chem. Soc.* **2019**, *141*, 5014–5020.

(14) Yang, L.; Wang, Y.; Chen, Y.; Yang, J.; Wang, X.; Li, L.; Li, J. Microporous Metal-Organic Framework with Specific Functional Sites for Efficient Removal of Ethane from Ethane/Ethylene Mixtures. *Chem. Eng. J.* **2020**, *387*, 124137.

(15) He, C.; Wang, Y.; Chen, Y.; Wang, X.; Yang, J.; Li, L.; Li, J. Direct Functionalization of the Open Metal Sites in Rare Earth-Based Metal–Organic Frameworks Used for the Efficient Separation of Ethylene. *Ind. Eng. Chem. Res.* **2020**, *59*, 6123–6129.

(16) Pires, J.; Fernandes, J.; Dedecker, K.; Gomes, J. R. B.; Pérez-Sánchez, G.; Nouar, F.; Serre, C.; Pinto, M. L. Enhancement of Ethane Selectivity in Ethane-Ethylene Mixtures by Perfluoro Groups in Zr-Based Metal-Organic Frameworks. *ACS Appl. Mater. Interfaces* **2019**, *11*, 27410–27421.

(17) Lee, S. K.; Lee, Y. J.; Cho, K.; Lee, U. H.; Chang, J. S. A Fluorinated Metal-Organic Framework, FMOF-2, for Preferential Adsorption of Ethane over Ethylene. *Bull. Korean Chem. Soc.* **2021**, *42*, 286–289.

(18) Chen, C. X.; Wei, Z. W.; Pham, T.; Lan, P. C.; Zhang, L.; Forrest, K. A.; Chen, S.; Al-Enizi, A. M.; Nafady, A.; Su, C. Y.; Ma, S. Nanospace Engineering of Metal–Organic Frameworks through Dynamic Spacer Installation of Multifunctionalities for Efficient

Separation of Ethane from Ethane/Ethylene Mixtures. *Angew. Chemie - Int. Ed.* **2021**, *60*, 9680–9685.

(19) Kang, M.; Yoon, S.; Ga, S.; Kang, D. W.; Han, S.; Choe, J. H.; Kim, H.; Kim, D. W.; Chung, Y. G.; Hong, C. S. High-Throughput Discovery of $Ni(IN)_2$ for Ethane/Ethylene Separation. *Adv. Sci.* **2021**, *8*, 2–8.

(20) Ding, Q.; Zhang, Z.; Zhang, P.; Yu, C.; He, C. H.; Cui, X.; Xing, H. One-Step Ethylene Purification from Ternary Mixture by Synergetic Molecular Shape and Size Matching in a Honeycomb-like Ultramicroporous Material. *Chem. Eng. J.* **2022**, *450*, 138272.

(21) Wang, S. M.; Wang, F.; Dong, Y. L.; Shivanna, M.; Dong, Q.; Mu, X. T.; Duan, J.; Yang, Q.; Zaworotko, M. J.; Yang, Q. Y. Reversed C_2H_6/C_2H_4 Separation in Interpenetrated Diamondoid Coordination Networks with Enhanced Host–Guest Interaction. *Sep. Purif. Technol.* **2021**, *276*, 119385.

(22) Zhang, Y.; Lv, D.; Chen, J.; Liu, Z.; Duan, C.; Chen, X.; Yuan, W.; Xi, H.; Xia, Q. Preferential Adsorption of Ethane over Ethylene on a Zr-Based Metal-Organic Framework: Impacts of C-H···N Hydrogen Bonding. *New J. Chem.* **2021**, 8045–8053.
